# Association between testosterone and cancers risk in women: a two-sample Mendelian randomization study

**DOI:** 10.1007/s12672-023-00811-2

**Published:** 2023-11-04

**Authors:** Zhizhou Li, Maoyu Wang, Meimian Hua, Ziwei Wang, Yidie Ying, Zhensheng Zhang, Shuxiong Zeng, Huiqing Wang, Chuanliang Xu

**Affiliations:** Department of Urology, Shanghai Changhai Hospital, Naval Medical University, Shanghai, China

**Keywords:** Mendelian randomization, Testosterone, Bioavailable testosterone, Cancer, Female

## Abstract

**Objective:**

Previous observational studies have explored the correlation between testosterone and cancer risk. However, the causal association between testosterone and various cancer types in women remains inconclusive. The objective of this Mendelian randomization study is to evaluate the causal links between total testosterone (TT) and bioavailable testosterone (BT) with cancer risk in females.

**Methods:**

Initially, a rigorous quality control process was employed to identify suitable instrumental single nucleotide polymorphisms (SNPs) associated with the exposure under investigation that exhibited a significant association. The genetic causal relationship between female testosterone levels and the risk of developing cancers was examined through a two-sample Mendelian randomization. Various analytical methods, including inverse-variance weighted (IVW), MR-Egger, weighted median, simple mode, and weighted mode, were applied in the investigation. Key findings were primarily based on the results obtained via IVW (random effects), and sensitivity analyses were conducted to assess the reliability of the obtained results. Furthermore, maximum likelihood, penalized weighted median, and IVW (fixed effects) methods were utilized to further validate the robustness of the results.

**Results:**

Based on the results of IVW analysis, our study indicated a positive causal relationship between BT and breast cancer (OR = 1.1407, 95%CI: 1.0627–1.2244, P = 0.0015) and endometrial cancer (OR = 1.4610, 95%CI: 1.2695–1.6813, P = 1.22E-06). Moreover, our findings also showed a positive causal association between TT and breast cancer (OR = 1.1764, 95%CI: 1.0846–1.2761, P = 0.0005), cervical cancer(OR = 1.0020, 95%CI: 1.0007–1.0032, P = 0.0077), and endometrial cancer(OR = 1.4124, 95%CI: 1.2083–1.6511, P = 0.0001). Additionally, our results demonstrated a negative causal relationship between BT and ovarian cancer (OR = 0.8649, 95%CI: 0.7750–0.9653, P = 0.0320). However, no causal relationship was found between BT, TT and other types of cancer (corrected P > 0.05).

**Conclusions:**

This study elucidates the role of testosterone on the development of breast cancer, endometrial cancer, ovarian cancer, and cervical cancer. It also hints at a potential but fragile link between testosterone and bladder cancer, as well as thyroid cancer. Nonetheless, it's worth noting that no statistically significant relationship between testosterone and various other types of cancer in females was identified.

**Supplementary Information:**

The online version contains supplementary material available at 10.1007/s12672-023-00811-2.

## Background

Testosterone is a hormone primarily synthesized in the testes, adrenal cortex, and ovaries, exerting various physiological effects on the body [1]. Total testosterone (TT) can exist in multiple forms, including sex hormone-binding globulin (SHBG)-bound testosterone, albumin-bound testosterone, corticosteroid-binding globulin (CBG)-bound testosterone, and unbound or free testosterone, each with different degrees of biological activity. SHBG-bound testosterone, due to its high binding affinity, displays limited bioavailability, while the non-SHBG-bound form, known as bioavailable testosterone (BT), is more physiologically active [2]. In men, testosterone plays a crucial role in the maintenance of muscular integrity, bone mineral density, and sexual function, with its deficiency potentially result in issues such as infertility, depression, fatigue, and decreased libido [3]. Despite being present at lower levels in women, testosterone exhibits greater sensitivity in females [4]. Emerging evidence suggests that testosterone levels may be correlated with suicidal behavior in females with bipolar disorder, and bioavailable testosterone is associated with the severity of COVID-19 infection in women [5, 6]. The influence of testosterone on the risk of cancer has been investigated in previous studies, but the results have been inconclusive. In a reanalysis of nine prospective studies, it was found that the levels of testosterone examined were significantly associated with an increased risk of breast cancer [7]. Conversely, another systematic review and meta-analysis of prospective studies suggested that circulating levels of SHBG and testosterone may affect the risk of gastric, liver, and colorectal cancer, but not in women [8]. Additionally, a prospective cohort study indicated that a doubling in female testosterone levels was associated with multifactorially adjusted hazard ratios of 2.21 (1.39–3.51; P = 0.001) for bladder cancer and 2.11 (1.09–2.32; P = 0.04) for liver cancer. On the other hand, the hazard ratios for pancreas and larynx cancer were 0.58 (0.35–0.97; P = 0.04) and 0.36 (0.15–0.90; P = 0.03) respectively, indicating a potential protective effect [9]. Therefore, the causal relationship between testosterone and the development of cancer remains uncertain and warrants further studies.

Mendelian randomization (MR) is an approach within genetic epidemiology that utilizes genetic variants as instrumental variables (IVs) to evaluate the causal relationships between risk factors and diseases [10]. The technique aims to address the limitations of traditional epidemiological studies by leveraging the random assortment of alleles during conception to overcome confounding and reverse causation [11]. There is substantial evidence supporting the reliability of MR, exemplified by a study by Wu et al. [12],which confirmed a causal link between bioavailable testosterone and sex hormone binding globulin in female primary biliary cholangitis. Therefore, MR analysis becomes essential in exploring the specific association between testosterone and cancers risk.

In this two-sample MR study, we employed expansive genome-wide association study (GWAS) datasets to examine the prospective causal relationships between testosterone levels and ten prevalent types of cancer in females. Through this investigation, our goal is to uncover the genetic features and biological pathways underlying the impact of testosterone on women's cancers risk.

## Material and methods

### Data collection

In the assessment of testosterone levels in women, both Total Testosterone (TT) and Bioavailable Testosterone (BT) are used as indicators for the physiological effects of testosterone. Moreover, we defined the ten cancers using the ICD-10 codes (Supplementary Table 1). For the purpose of this study, individuals of European ancestry were recruited for inclusion. Pertinent data related to individual samples, such as sample size, demographic characteristics, and the number of relevant single nucleotide polymorphisms (SNPs), can be found in Supplementary Table 2. Summary statistics for multiple samples were sourced from the Medical Research Council Integrative Epidemiology Unit Open GWAS project (https://gwas.mrcieu.ac.uk/). Due to our exclusive use of deidentified data, institutional review board approval was not required for our analysis.

Data concerning Total Testosterone (TT), Bioavailable Testosterone (BT), cervical cancer, endometrial cancer, bladder cancer, and thyroid cancer were exclusively sourced from the MRC IEU Open GWAS database. Liver and bile duct cancer data was obtained from the Genome-wide Association Study of Cancer Risk in the UK Biobank. Publicly available summary statistics from the FinnGen consortium (www.finbb.fi) provided data on kidney cancer and stomach cancer. Additionally, Neale Lab contributed data on skin cancer. Finally, data on breast cancer and ovarian cancer were respectively obtained from the Breast Cancer Association Consortium (BCAC) and the Ovarian Cancer Association Consortium (OCAC). The GWAS datasets for 10 different types of cancer were presented in Table 1.

**Table 1:** The GWAS datasets for 10 different types of cancer

GWAS_ID	Disease	Consortium	Case	Control	Sample size	Population
ieu-a-1126	Breast cancer	BCAC	122,977	105,974	228,951	European
ieu-a-1120	Ovarian cancer	OCAC	25,509	40,941	66,450	European
ieu-b-4876	Cervical cancer	NA	563	198,523	199,086	European
ebi-a-GCST006464	Endometrial cancer	NA	12,906	108,979	121,885	European
ukb-b-8193	Bladder cancer	MRC-IEU	1,101	461,832	462,933	European
finn-b-C3_KIDNEY_NOTRENALPELVIS	Kidney cancer	Finngen	971	217,821	NA	European
ieu-a-1082	Thyroid cancer	NA	649	431	1,080	European
finn-b-C3_STOMACH	Stomach cancer	Finngen	633	218,159	NA	European
ieu-b-4915	Liver and bile duct cancer	UK Biobank	350	372,016	372,366	European
ukb-d-C_SKIN	Cancer of skin	Neale lab	16,531	344,663	361,194	European

### Instrumental variable selection

**Fig. 1:**
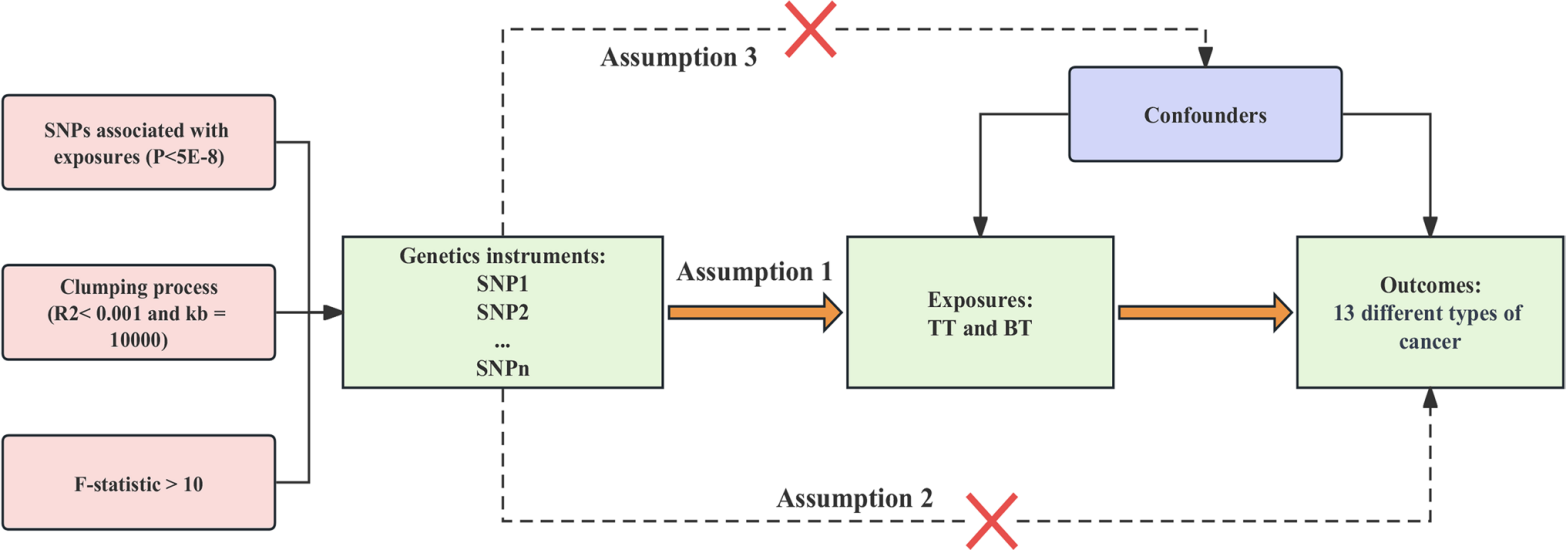
The study design and three fundamental assumptions in the Mendelian randomization analysis

In this study, we utilized the genetic variants (TT and BT) associated with exposures as instrumental variables (IVs) satisfying three assumptions, namely: (1) the genetic variants are associated with exposures, (2) the genetic variants solely influence outcomes through the exposures, and (3) the genetic variants are unrelated to any confounding factors affecting the exposure-outcome association [13]. We identified SNPs that exhibited significant associations with the exposures (P < 5E-8) and ensured their independence by assessing linkage disequilibrium (LD). To eliminate LD among the SNPs, clumping process was implemented with R2 < 0.001 and kb = 10,000. The F statistics were calculated using the formula (F = β2/SE2) for each SNP separately [14, 15], Subsequently, we confirmed that the F statistic exceeded 10 in order to mitigate any potential effects of weak instrumental variable bias. Figure 1 depicted the study design through a study framework diagram. The PhenoScanner database (http://www.phenoscanner.medschl.cam.ac.uk) was employed to scrutinize all SNPs. Supplementary Tables 3 and 4 respectively displayed the characteristics of IVs associated with BT and TT, excluding the influence of confounding factors such as body mass index, smoking, diabetes, and the use of blood pressure medication. Supplementary Tables 5 and 6 presented the distinct IVs selected for BT and TT in conducting MR analysis to assess the risk of multiple cancers in cancer-specific GWAS.

### Mendelian randomization statistical analysis

The Wald ratio approach was employed to estimate the impact of a single SNP closely linked with exposure on the outcome, with the inverse-variance weighted (IVW, random effects) serving as the primary analytical method. Supplementary analyses were conducted using MR-Egger, weighted median, simple mode, and weighted mode. When all included SNPs are effective instrumental variables, the IVW method was utilized for precise estimation [16]. The MR-Egger method adjusted for pleiotropy, yet the resulting associations tend to be less precise [17]. The weighted median model is a valid estimator when at least 50% of the weight is derived from appropriate instrumental variables [18]. Heterogeneity and horizontal pleiotropy were assessed using Cochrane's Q and MR-Egger intercept, respectively [17]. In cases where there is no excess heterogeneity, the random-effects IVW models and the fixed-effect IVW model yield equivalent results without a loss of precision. Conversely, random-effects IVW models are appropriate when heterogeneity is present [19]. If there is evidence of horizontal pleiotropy, the MR-PRESSO outlier test was applied [20]. Leave-one-out sensitivity analysis was additionally used to identify SNPs with influential effects and assess the reliability of the results [21]. To further demonstrate the reliability of MR results, maximum likelihood, penalized weighted median, and IVW (fixed effects) were utilized in the subsequent analyses. The maximum likelihood approach is a conventional method with low standard errors similar to IVW and, if the hypotheses are met, yields unbiased results with smaller standard errors than IVW [22].

All of our statistical tests were two-sided, and a P < 0.05 was considered statistically significant. All data analysis was implemented using R Studio 4.2.1 with the “Two-Sample-MR”, “MR-PRESSO” and “MendelianRandomization” packages for MR analysis.

## Results

### 1Mendelian randomization analysis

**Fig. 2:**
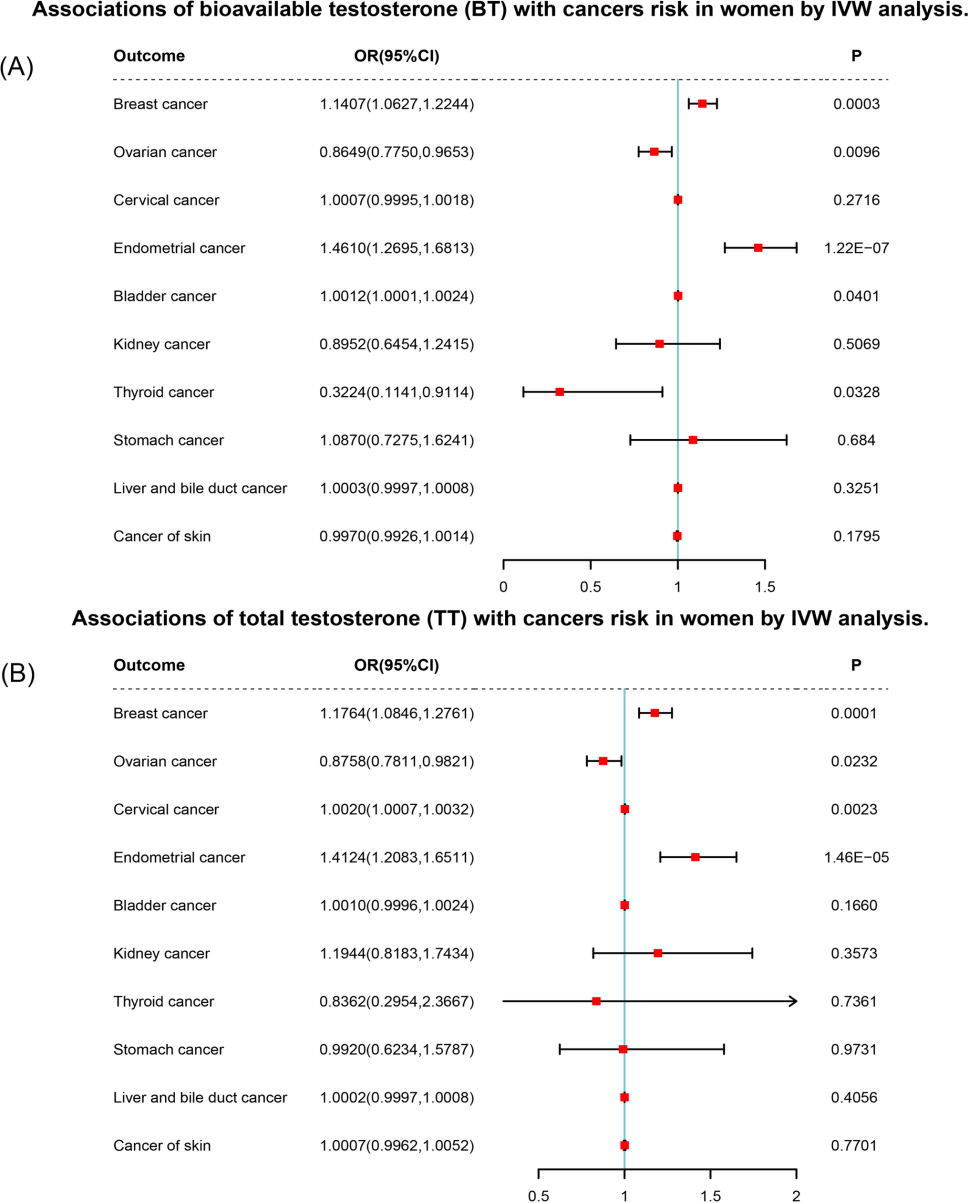
Forest plots of two-sample Mendelian randomization (MR) estimation of the associations of **A** BT and **B** TT with cancers risk in women. The odds ratio (OR) was estimated using inverse variance weighting (IVW). CI, confidence interval

Through screening and extraction of SNPs based on the aforementioned criteria, we identified 106 and 113 candidate instrumental variables (IVs) for TT and BT, respectively, using various multiple testing strategies such as LD. All corrected P-value of IVW presented below were calculated using the false discovery rate (FDR) method. By employing IVW analysis, we revealed that BT exhibited a positive causal association with breast cancer (OR = 1.1407, 95%CI: 1.0627–1.2244, P = 0.0015) and endometrial cancer (OR = 1.4610, 95%CI: 1.2695–1.6813, P = 1.22E-06). Moreover, IVW analysis also supported a positive causal relationship between TT and breast cancer (OR = 1.1764, 95%CI: 1.0846–1.2761, P = 0.0005), cervical cancer (OR = 1.0020, 95%CI: 1.0007–1.0032, P = 0.0077), and endometrial cancer (OR = 1.4124, 95%CI: 1.2083–1.6511, P = 0.0001). Furthermore, our results demonstrated a negative causal relationship between BT and ovarian cancer (OR = 0.8649, 95%CI: 0.7750–0.9653, P = 0.0320). Additionally, it is imperative to acknowledge that the findings prior to P-value adjustment should not be disregarded. We discovered a positive causal correlation between BT and bladder cancer (OR = 1.0012, 95%CI: 1.0001–1.0024, P = 0.0401), while BT and TT respectively exhibited a negative causal association with thyroid cancer (OR = 0.3224, 95%CI: 0.1141–0.9114, P = 0.0328) and ovarian cancer (OR = 0.8758, 95%CI: 0.7811–0.9821, P = 0.0232). To sum up, our findings were primarily based on IVW analysis (Fig. 2 and Supplementary Tables 7 and 8).

### Sensitivity analysis

**Fig. 3:**
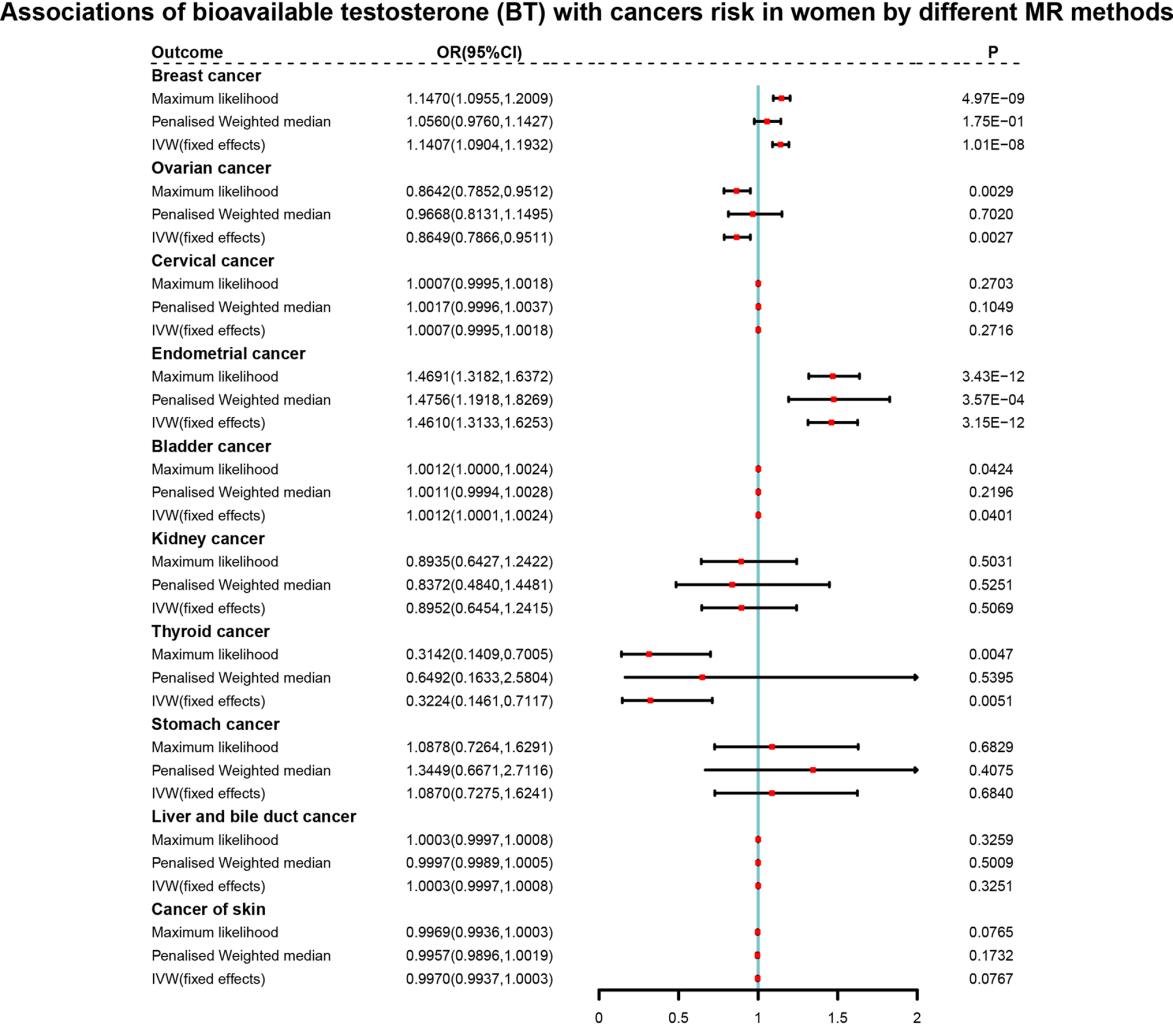
Forest plots of further validation of mendelian randomization (MR) analysis for the associations of bioavailable testosterone (BT) with cancers risk in women

**Fig. 4:**
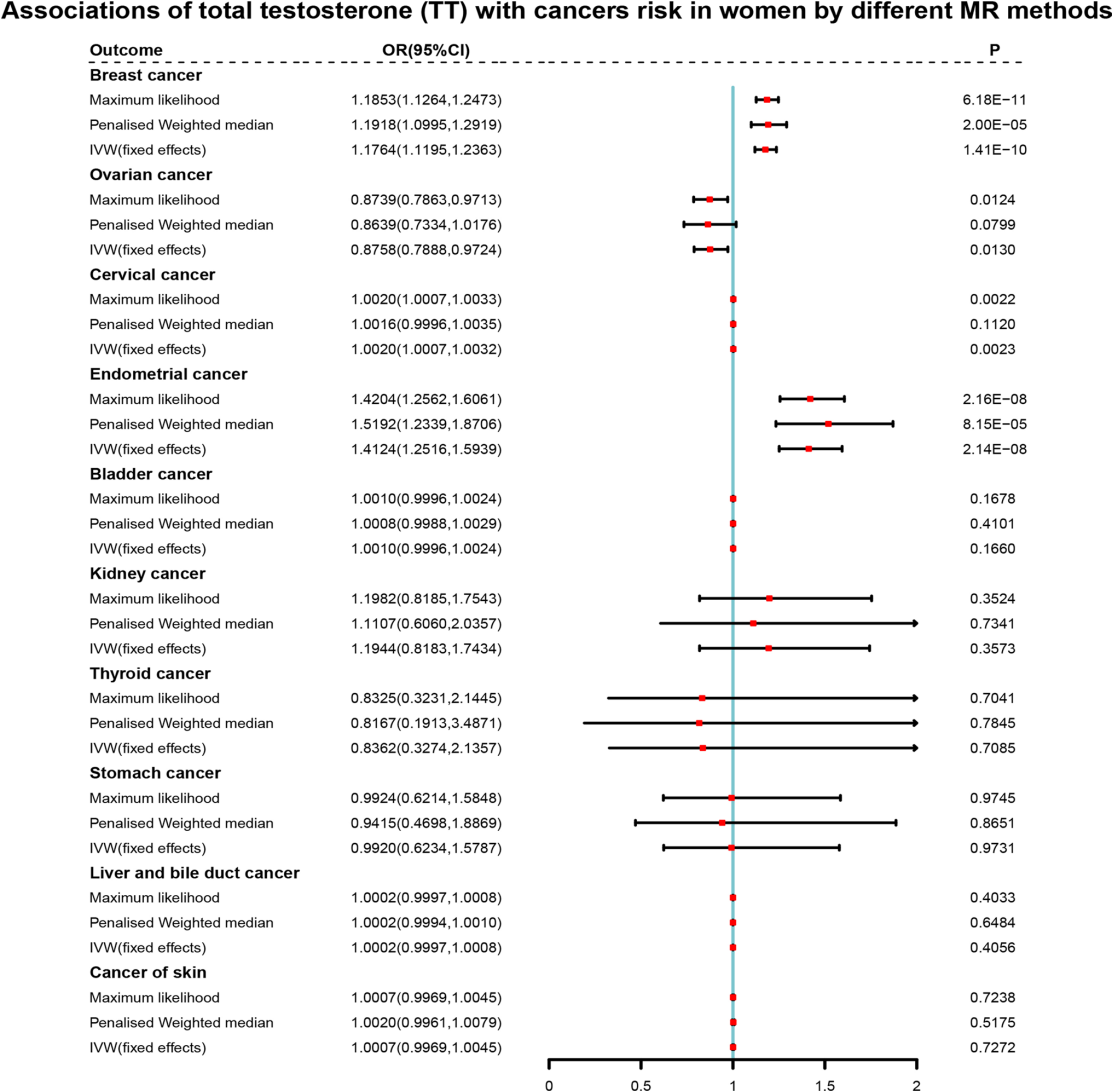
Forest plots of further validation of mendelian randomization (MR) analysis for the associations of total testosterone (TT) with cancers risk in women

In order to verify the reliability of the IVW results, sensitivity analysis was conducted. The IVW and MR-Egger tests for heterogeneity indicated the absence of heterogeneity in the MR analysis results between BT and bladder cancer (P > 0.05), as well as between TT and cervical and ovarian cancer (P > 0.05). However, heterogeneity was observed among BT across breast cancer, endometrial cancer, and thyroid cancer (P < 0.05), as well as between TT and breast cancer and endometrial cancer (P < 0.05) (Supplementary Tables 9 and 10). Since the IVW accounts for potential heterogeneity among the Wald ratio estimates from SNPs, the use of random-effects IVW models ensured results accuracy. All MR-Egger intercept tests showed P > 0.05, indicating the absence of horizontal pleiotropy. Nevertheless, the MR-PRESSO test was further applied to ensure the accuracy of the results. Additionally, although no single SNP had a significant influence on the MR estimation results according to the leave-one-out analysis, maximum likelihood, penalized weighted median, and IVW (fixed effects) were implemented to further verify the IVW results (Figs. 3,4 and Supplementary Tables 11, 12).

The scatter plot provides a more illustrative representation of the associations with BT and TT on cancers risk (Figs. 5, 6). Furthermore, forest plots for each pair of causal effects were presented in the additional files (Supplementary Figures: 1–9). The results from the Leave-one-out analysis suggested that there was no significant effect on the results (Supplementary Figures: 10–18).

**Fig. 5:**
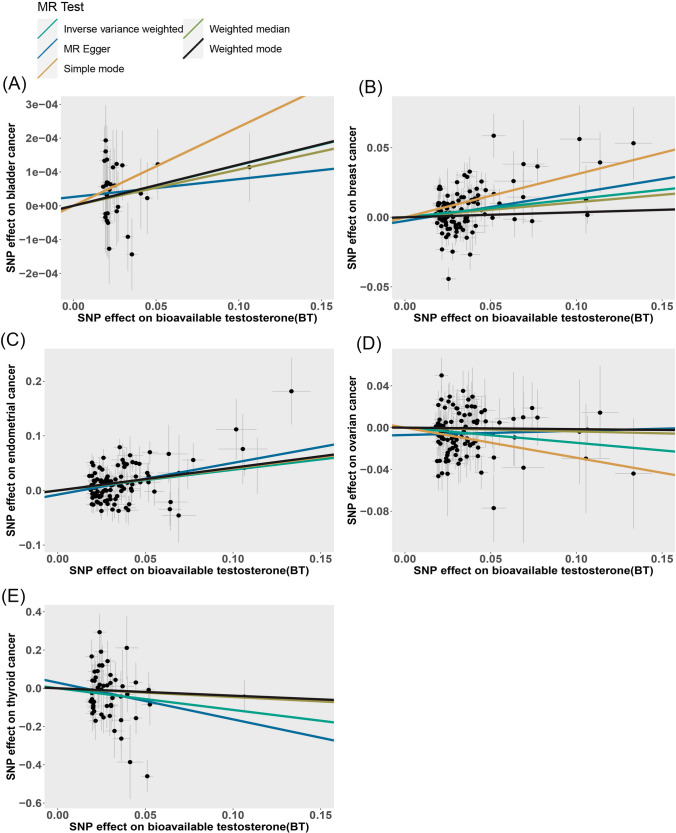
Scatter plots of the causal effect of bioavailable testosterone (BT) on **A** Bladder cancer, **B** Breast cancer, **C** Endometrial cancer, **D** Ovarian cancer and **E** thyroid cancer. The causal effect of each method is represented separately by the slopes of line

**Fig. 6:**
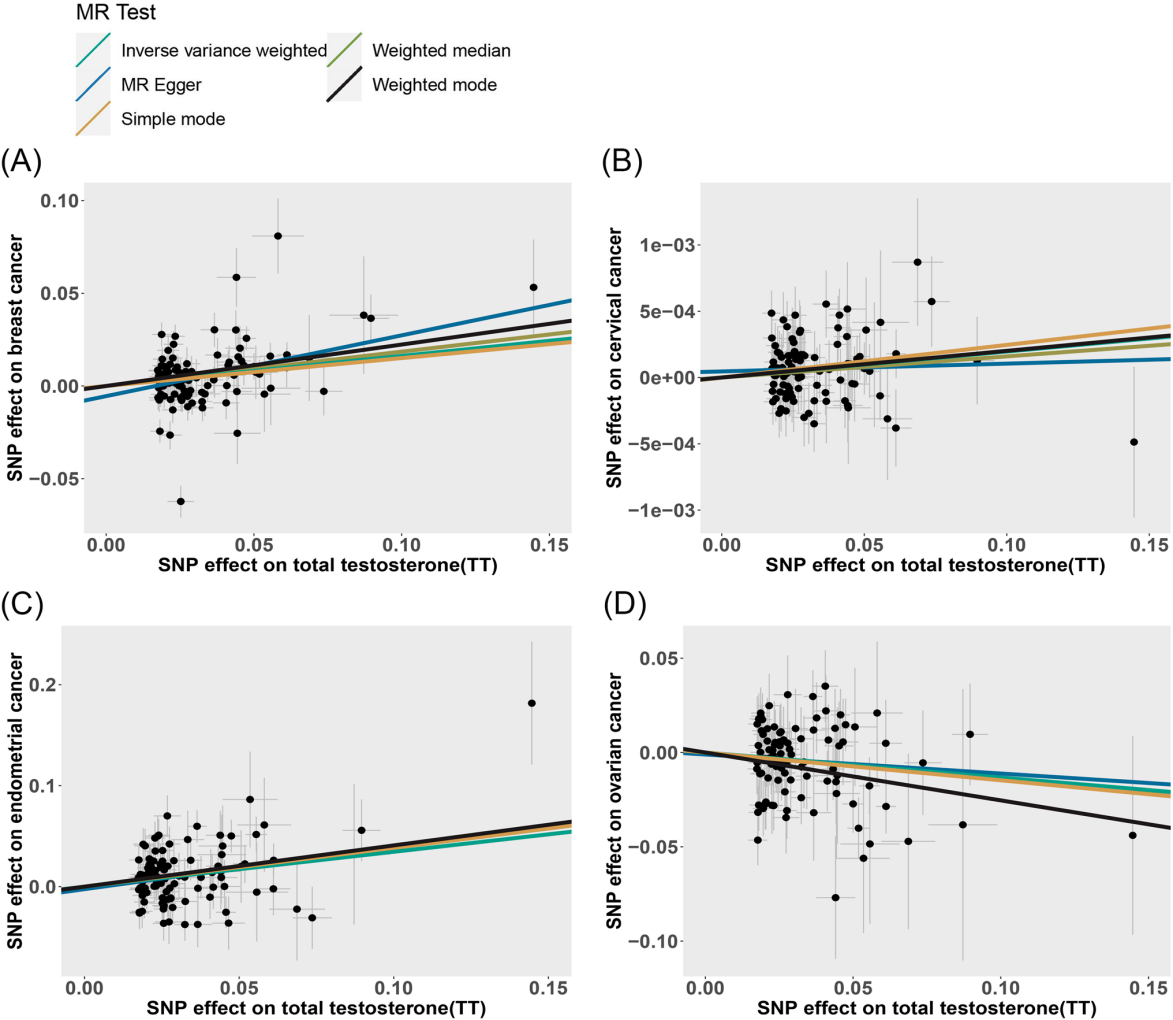
Scatter plots of the causal effect of total testosterone (TT) on **A** Breast cancer, **B** Cervical cancer, **C** Endometrial cancer and **D** Ovarian cancer. The causal effect of each method is represented separately by the slopes of line

## Discussion

This study employed a two-sample MR approach to investigate the correlation between female testosterone levels and the propensity for 10 specific malignancies in the European populace. The findings revealed a positive causal association between BT and breast cancer as well as endometrial cancer. Moreover, the analysis also exposed a positive causal association between TT and breast cancer, cervical cancer, and endometrial cancer. Furthermore, A negative causal relationship between BT and ovarian cancer was demonstrated. Additionally, the potential but tenuous associations between BT and bladder cancer, as well as thyroid cancer, should not be overlooked. However, no causative link was identified for renal, gastric, hepatic, biliary, or skin cancers.

Our findings are consistent with several prior studies that have identified a correlation between women's testosterone levels and the risk of certain cancers. Kenemans P et al. [23] have concluded that elevated serum testosterone levels in women are significantly associated with an increased risk of breast cancer development. Furthermore, there is evidence suggesting that androgens and the androgen receptor may contribute to endometrial cell proliferation by regulating expression of insulin growth factor I (IGF-I) [24]. Johnson, D.T. et al. [25] have demonstrated that transgenic androgen receptor expression promotes cellular proliferation of bladder cancer using different experimental manipulations of endogenous androgen levels. A prospective study of 126 European women indicated a significant positive correlation between testosterone levels and invasive cervical cancer risk in both pre- and postmenopausal women [26].

In addition, previous research has established significant causal links between women's testosterone levels and the risk of ovarian, thyroid, and lung cancers. Notably, a nested case–control study analyzing data from 7 studies conducted in North America and Europe involving 1,331 epithelial ovarian cancer cases and 3,017 matched controls found testosterone to be significantly positively correlated with epithelial ovarian cancer risk [27]. The distinct patterns of testosterone and androgen receptor expression patterns observed in the thyroid tissues of men and women could contribute to the gender-specific incidence of thyroid tumors [28]. In a case–control study, high levels of circulating free testosterone were negatively associated with the risk of lung cancer in postmenopausal never-smoking women [29].Nevertheless, our findings indicated a negative correlation between women's testosterone levels and the risk of ovarian and thyroid cancers. A previous extensive prospective, nested case–control study suggested no clear causal relationship between testosterone and ovarian cancer risk. However, in elderly women, there were suggestive negative associations between dehydroepiandrosterone or dehydroepiandrosterone sulfate levels and ovarian cancer risk [30]. Furthermore, the activation of the androgen receptor (AR) triggers cellular senescence in thyroid cancer cells [31]. Elevated androgen-AR expression suppresses the migratory and invasive properties of thyroid cancer cells, implying a potential anti-tumor effect of testosterone in thyroid cancer [32]. These findings should undergo further validation through additional observational studies.

In premenopausal females, peak serum testosterone and free testosterone levels were observed during midcycle and remained elevated during the mid luteal phase. Conversely, postmenopausal females exhibited significantly lower levels of both testosterone markers compared to premenopausal females [33]. Recent research by Ørsted, D.D. et al. [9] has indicated that increased levels of plasma testosterone in both males and females are correlated with a 30% to 80% heightened risk of premature mortality following a cancer diagnosis. Our hypothesis posits that elevated testosterone levels may trigger intracellular protein synthesis, which consequently contributes to the accelerated proliferation of both healthy and cancerous cells.

The current study is not without limitations. Foremost, this study solely assessed data from the European population, hence warranting caution when generalizing the findings to other ethnicities in forthcoming research. Furthermore, due to limitations in sample size from public databases, certain cancers were excluded in order to ensure the reliability of the research findings. Lastly, due to insufficient secondary data, a stratified analysis could not be conducted in this study.

## Conclusions

Conclusively, this study is the most comprehensive MR analysis to infer causation in the correlation between women's testosterone levels and the risk of multiple cancers in the European population. The result indicate that BT is positively associated with a higher risk of breast and endometrial cancers. Similarly, TT shows a positive causal association with breast, cervical, and endometrial cancers. Moreover, a negative causal relationship between BT and ovarian cancer was also observed. Additionally, BT has a potential but tenuous link with bladder cancer and thyroid cancer, although further substantiation through future research endeavors is warranted.

### Supplementary Information

**Additional file 1:  Table S1.** ICD-10 diagnosis codes for ten cancers. **Table S2.** Summary of data source of different Traits. **Table S3.** Characteristics of bioavailable testosterone (BT) associated instrumental variants (IVs). **Table S4.** Characteristics of total testosterone (TT) associated instrumental variants (IVs). **Table S5.** The instrumental variants (IVs) selected for bioavailable testosterone (BT) to perform Mendelian randomization analysis with multiple cancers risk in cancer-specific GWAS. **Table S6.** The instrumental variants (IVs) selected for total testosterone (TT) to perform Mendelian randomization analysis with multiple cancers risk in cancer-specific GWAS. **Table S7.** Mendelian randomization (MR) analysis for the associations of bioavailable testosterone (BT) with cancers risk in cancer-specific GWAS. **Table S8.** Mendelian randomization (MR) analysis for the associations of total testosterone (TT) with cancers risk in cancer-specific GWAS. **Table S9.** Sensitivity analysis of mendelian randomization analysis of bioavailable testosterone (BT) on different types of cancer. **Table S10.** Sensitivity analysis of mendelian randomization analysis of total testosterone (TT) on different types of cancer. **Table S11.** Further validation of mendelian randomization (MR) analysis for the associations of bioavailable testosterone (BT) with cancers risk in cancer-specific GWAS. **Table S12.** Further validation of mendelian randomization (MR) analysis for the associations of total testosterone (TT) with cancers risk in cancer-specific GWAS.

**Additional file 2: Figure S1.** Forest plot of the causal effect of bioavailable testosterone (BT) on bladder cancer. **Figure S2.** Forest plot of the causal effect of bioavailable testosterone (BT) on breast cancer. **Figure S3.** Forest plot of the causal effect of bioavailable testosterone (BT) on endometrial cancer. **Figure S4.** Forest plot of the causal effect of bioavailable testosterone (BT) on ovarian cancer. **Figure S5.** Forest plot of the causal effect of bioavailable testosterone (BT) on thyroid cancer. **Figure S6.** Forest plot of the causal effect of total testosterone (TT) on breast cancer. **Figure S7.** Forest plot of the causal effect of total testosterone (TT) on cervical cancer. **Figure S8.** Forest plot of the causal effect of total testosterone (TT) on endometrial cancer. **Figure S9.** Forest plot of the causal effect of total testosterone (TT) on ovarian cancer. **Figure S10.** Leave-one-out analysis of bioavailable testosterone (BT) on bladder cancer. **Figure S11.** Leave-one-out analysis of bioavailable testosterone (BT) on breast cancer. **Figure S12.** Leave-one-out analysis of bioavailable testosterone (BT) on endometrial cancer. **Figure S13.** Leave-one-out analysis of bioavailable testosterone (BT) on ovarian cancer. **Figure S14.** Leave-one-out analysis of bioavailable testosterone (BT) on thyroid cancer. **Figure S15.** Leave-one-out analysis of total testosterone (TT) on breast cancer. **Figure 16.** Leave-one-out analysis of total testosterone (TT) on cervical cancer. **Figure S17.** Leave-one-out analysis of total testosterone (TT) on endometrial cancer. **Figure S18.** Leave-one-out analysis of total testosterone (TT) on ovarian cancer.

## Data Availability

The datasets produced and/or analyzed in this study can be obtained from the corresponding author upon reasonable request.
