# Supplementary material for: Association between testosterone and cancers risk in women: a two-sample Mendelian randomization study
Source: Discov Oncol. 2023 Nov 4;14:198. doi: 10.1007/s12672-023-00811-2 (PMC10625503; doi:10.1007/s12672-023-00811-2)
Supplement: Supplementary file 2 — Additional file 2: Figure S1. Forest plot of the causal effect of bioavailable testosterone (BT) on bladder cancer. Figure S2. Forest plot of the causal effect of bioavailable testosterone (BT) on breast cancer. Figure S3. Forest plot of the causal effect of bioavailable testosterone (BT) on endometrial cancer. Figure S4. Forest plot of the causal effect of bioavailable testosterone (BT) on ovarian cancer. Figure S5. Forest plot of the causal effect of bioavailable testosterone (BT) on thyroid cancer. Figure S6. Forest plot of the causal effect of total testosterone (TT) on breast cancer. Figure S7. Forest plot of the causal effect of total testosterone (TT) on cervical cancer. Figure S8. Forest plot of the causal effect of total testosterone (TT) on endometrial cancer. Figure S9. Forest plot of the causal effect of total testosterone (TT) on ovarian cancer. Figure S10. Leave-one-out analysis of bioavailable testosterone (BT) on bladder cancer. Figure S11. Leave-one-out analysis of bioavailable testosterone (BT) on breast cancer. Figure S12. Leave-one-out analysis of bioavailable testosterone (BT) on endometrial cancer. Figure S13. Leave-one-out analysis of bioavailable testosterone (BT) on ovarian cancer. Figure S14. Leave-one-out analysis of bioavailable testosterone (BT) on thyroid cancer. Figure S15. Leave-one-out analysis of total testosterone (TT) on breast cancer. Figure 16. Leave-one-out analysis of total testosterone (TT) on cervical cancer. Figure S17. Leave-one-out analysis of total testosterone (TT) on endometrial cancer. Figure S18. Leave-one-out analysis of total testosterone (TT) on ovarian cancer. [file 12672_2023_811_MOESM2_ESM.docx]

**Association between testosterone and cancers risk in women: a two-sample Mendelian randomization study**

Zhizhou Li^1#^,Maoyu Wang^1#^,Meimian Hua^1#^,Ziwei Wang^1^,Yidie Ying^1^,Zhensheng Zhang^1^,Shuxiong Zeng^1^*****,Huiqing Wang^1^*****,Chuanliang Xu^1^*****.

Supplementary Figure.1

Supplementary Figure.2

Supplementary Figure.3

Supplementary Figure.4

Supplementary Figure.5

Supplementary Figure.6

Supplementary Figure.7

Supplementary Figure.8

Supplementary Figure.9

Supplementary Figure.10

Supplementary Figure.11

Supplementary Figure.12

Supplementary Figure.13

Supplementary Figure.14

Supplementary Figure.15

Supplementary Figure.16

Supplementary Figure.17

Supplementary Figure.18


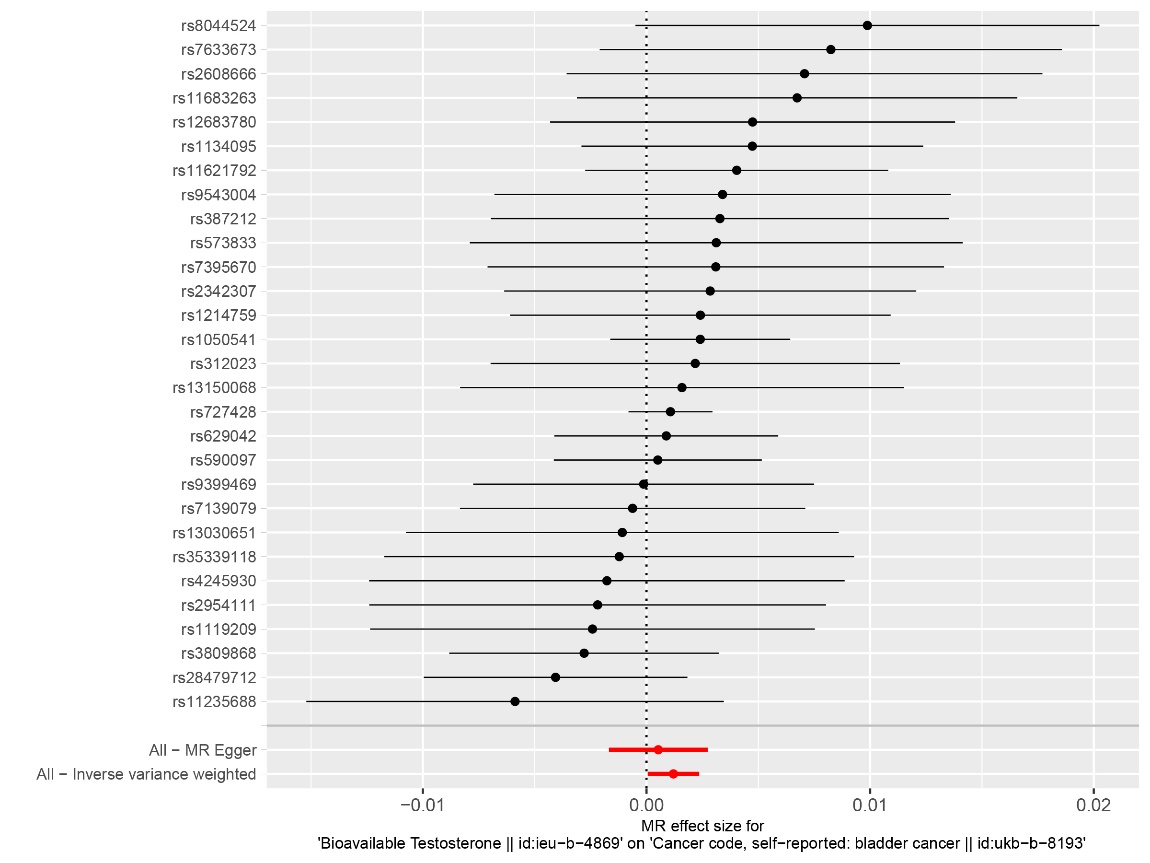


Supplementary Figure.1 Forest plot of the causal effect of bioavailable testosterone (BT) on bladder cancer.


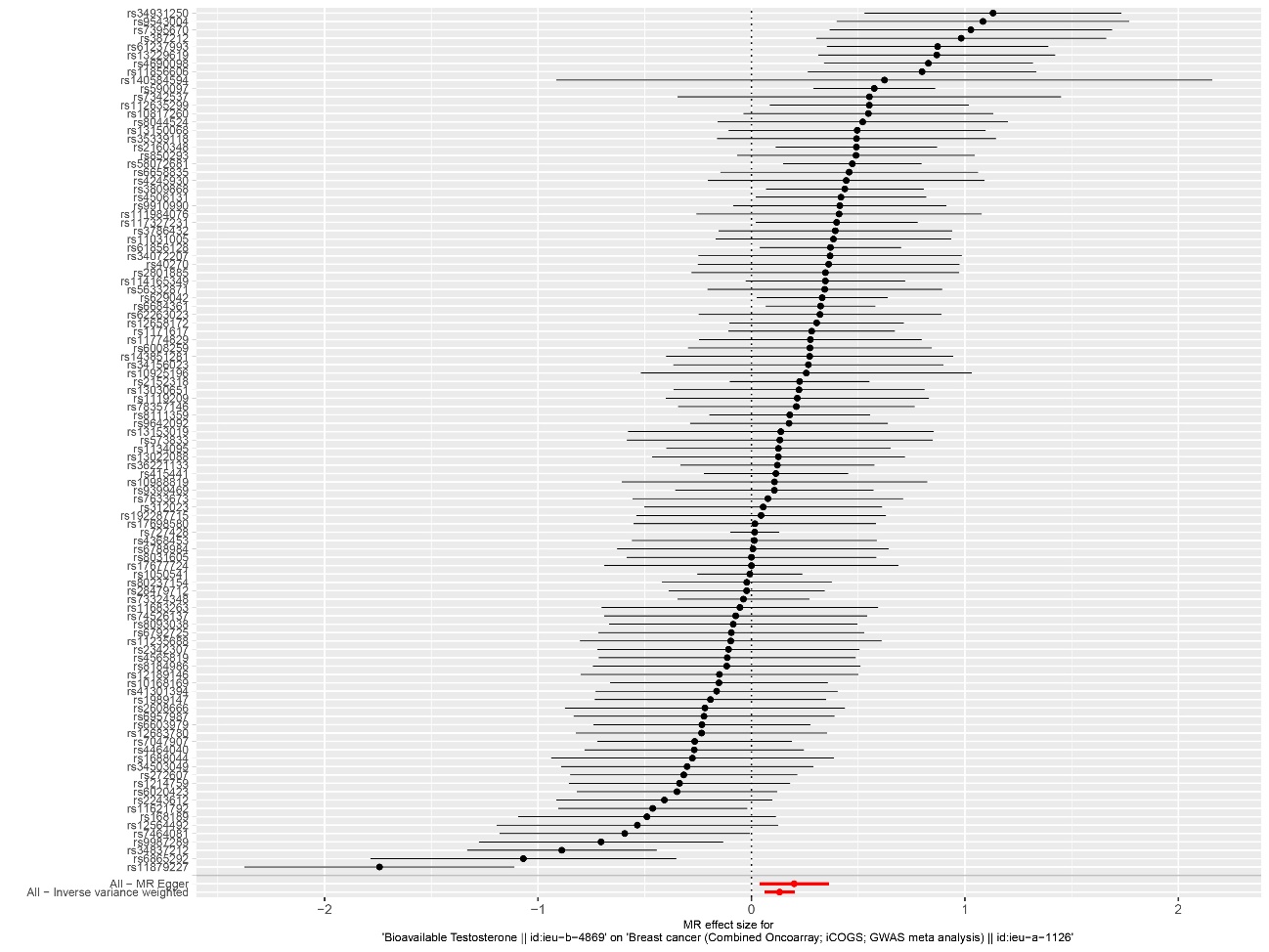


Supplementary Figure.2 Forest plot of the causal effect of bioavailable testosterone (BT) on breast cancer.


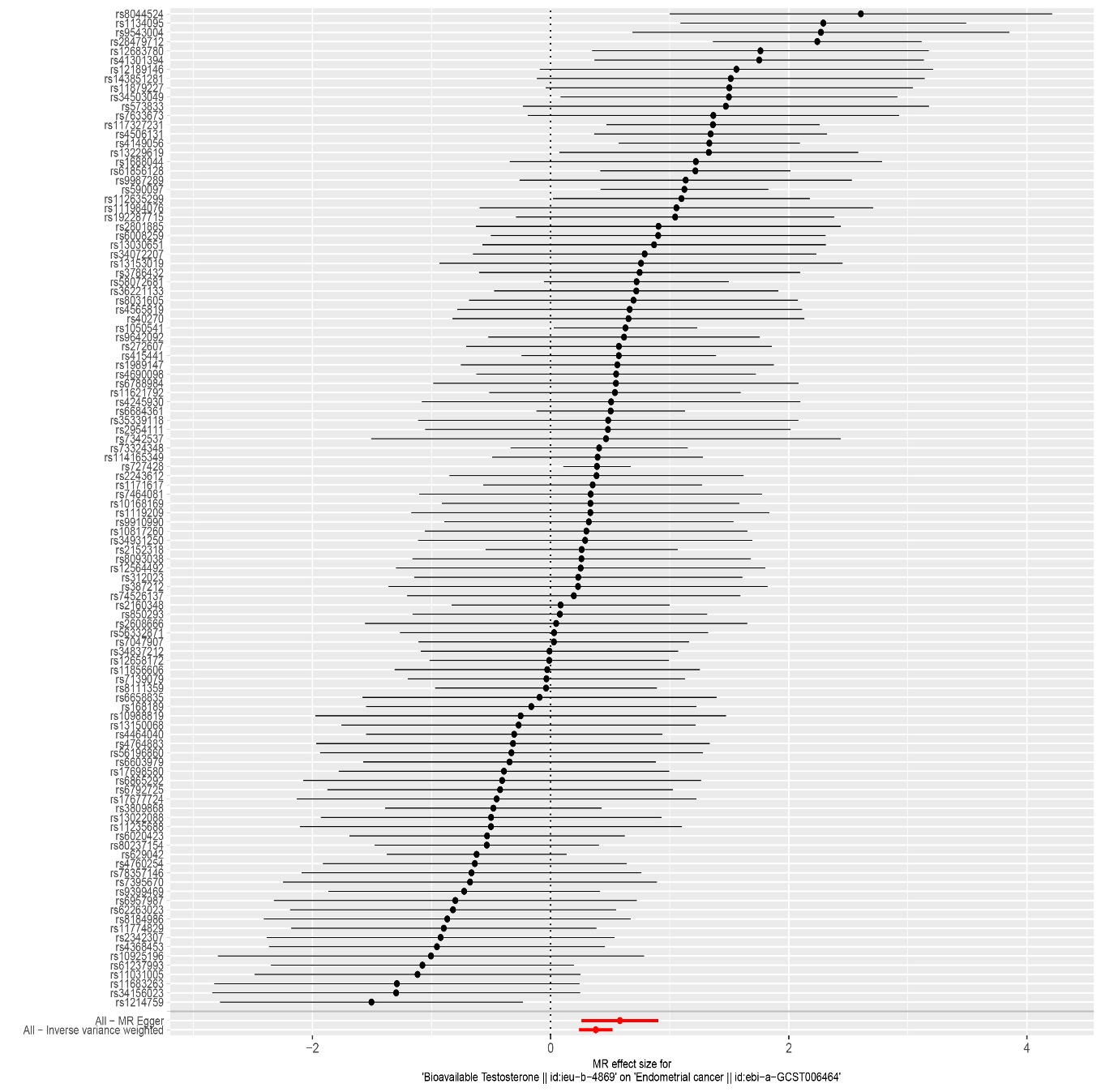


Supplementary Figure.3 Forest plot of the causal effect of bioavailable testosterone (BT) on endometrial cancer.


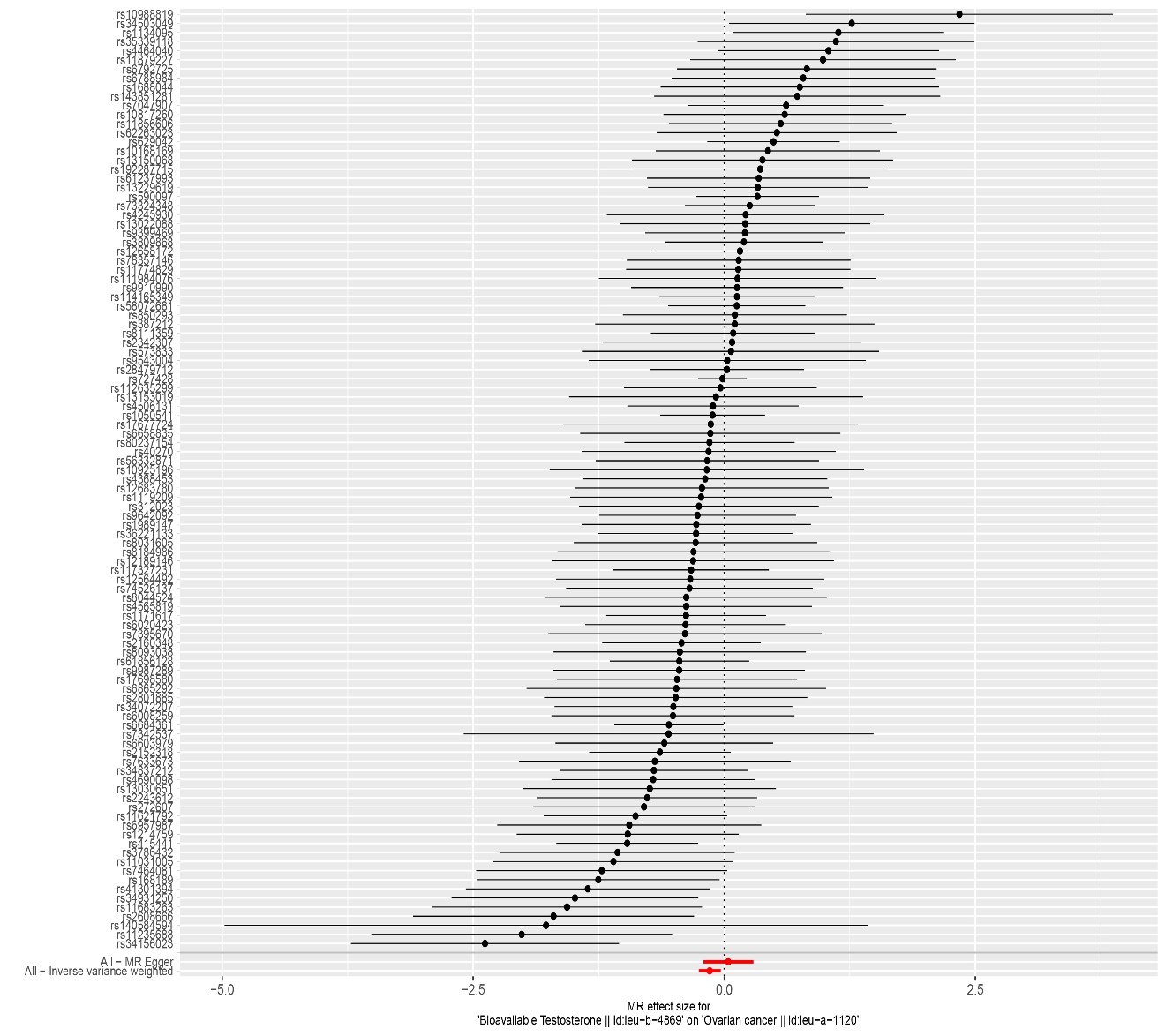


Supplementary Figure.4 Forest plot of the causal effect of bioavailable testosterone (BT) on ovarian cancer.


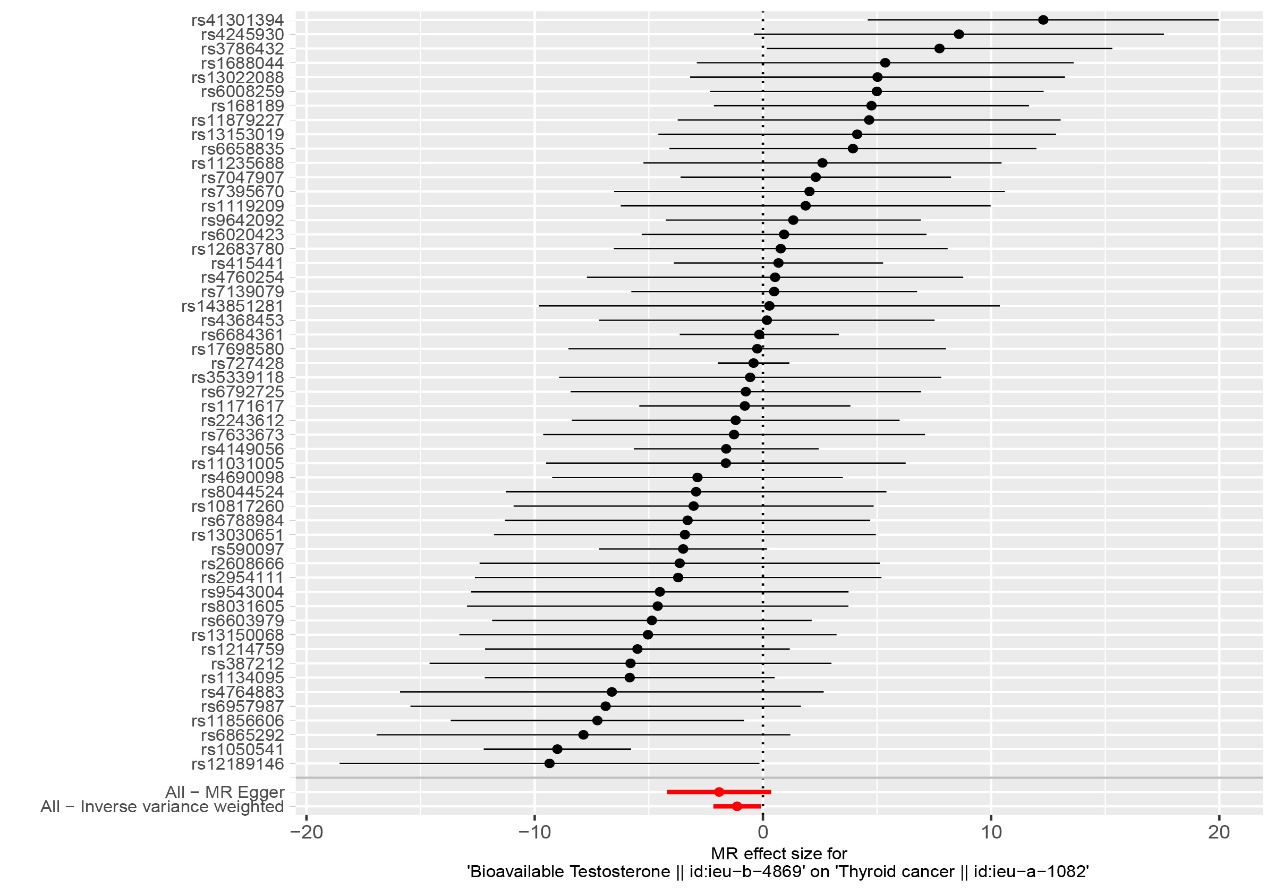


Supplementary Figure.5 Forest plot of the causal effect of bioavailable testosterone (BT) on thyroid cancer.


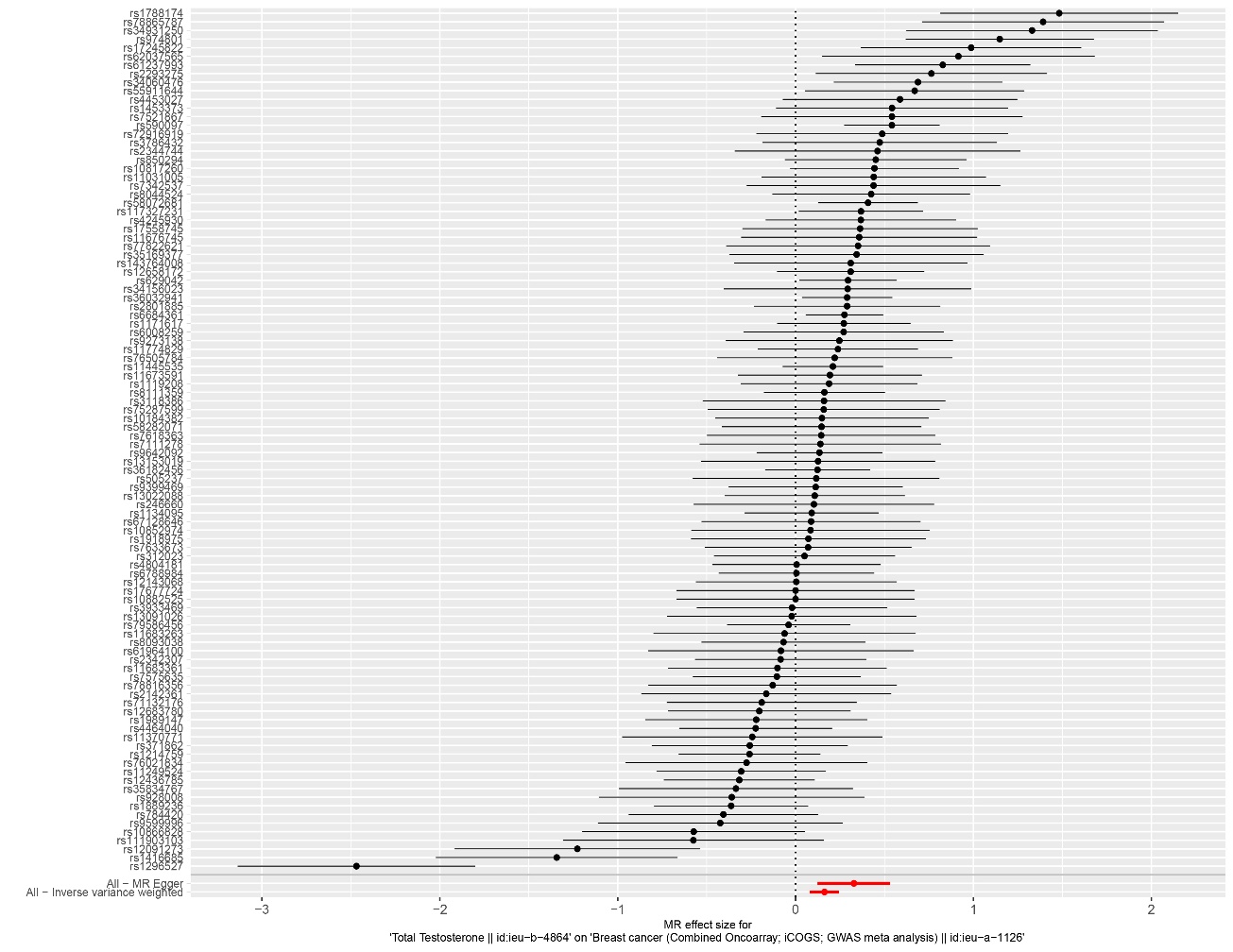


Supplementary Figure.6 Forest plot of the causal effect of total testosterone (TT) on breast cancer.


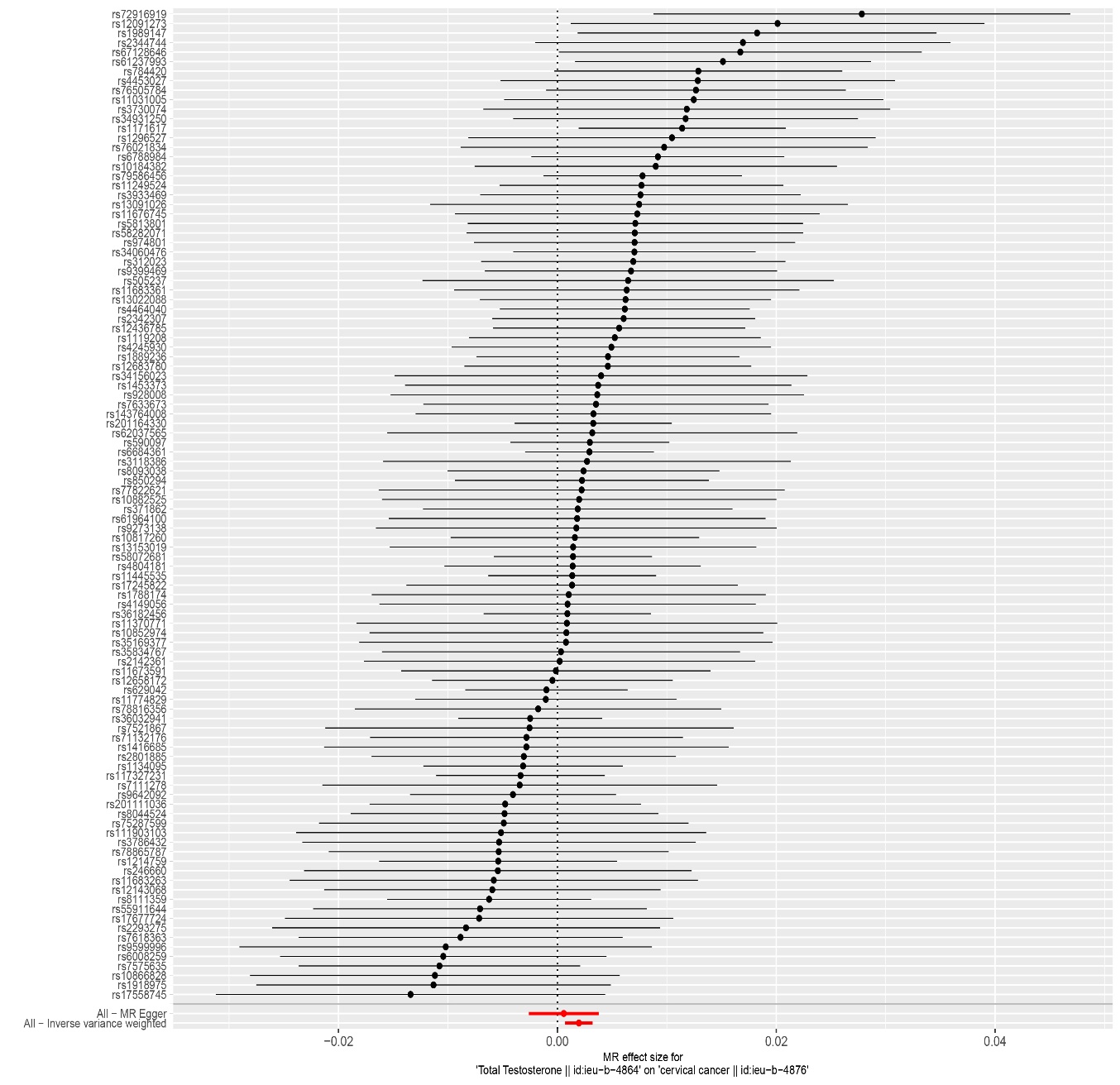


Supplementary Figure.7 Forest plot of the causal effect of total testosterone (TT) on cervical cancer.


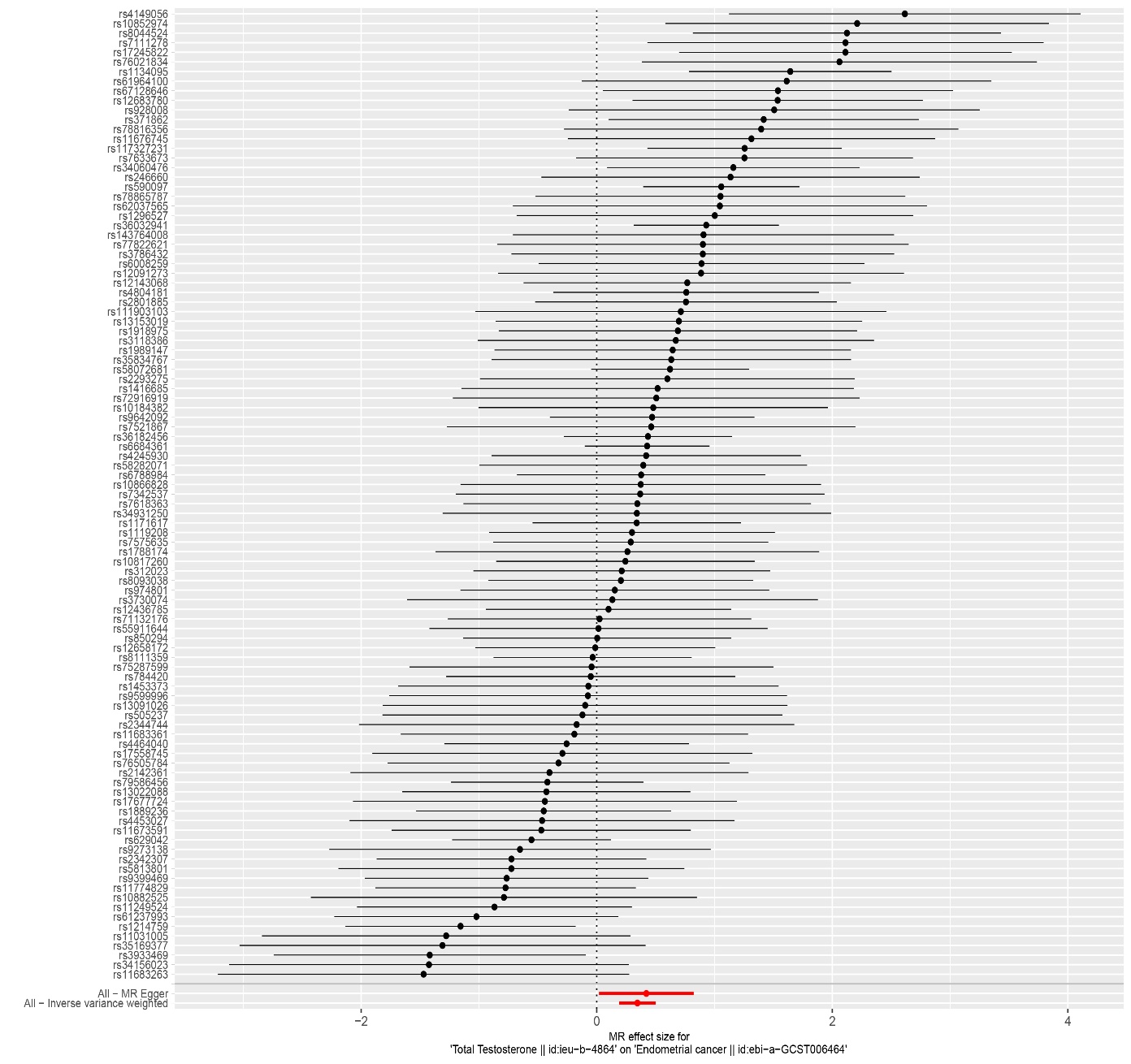


Supplementary Figure.8 Forest plot of the causal effect of total testosterone (TT) on endometrial cancer.


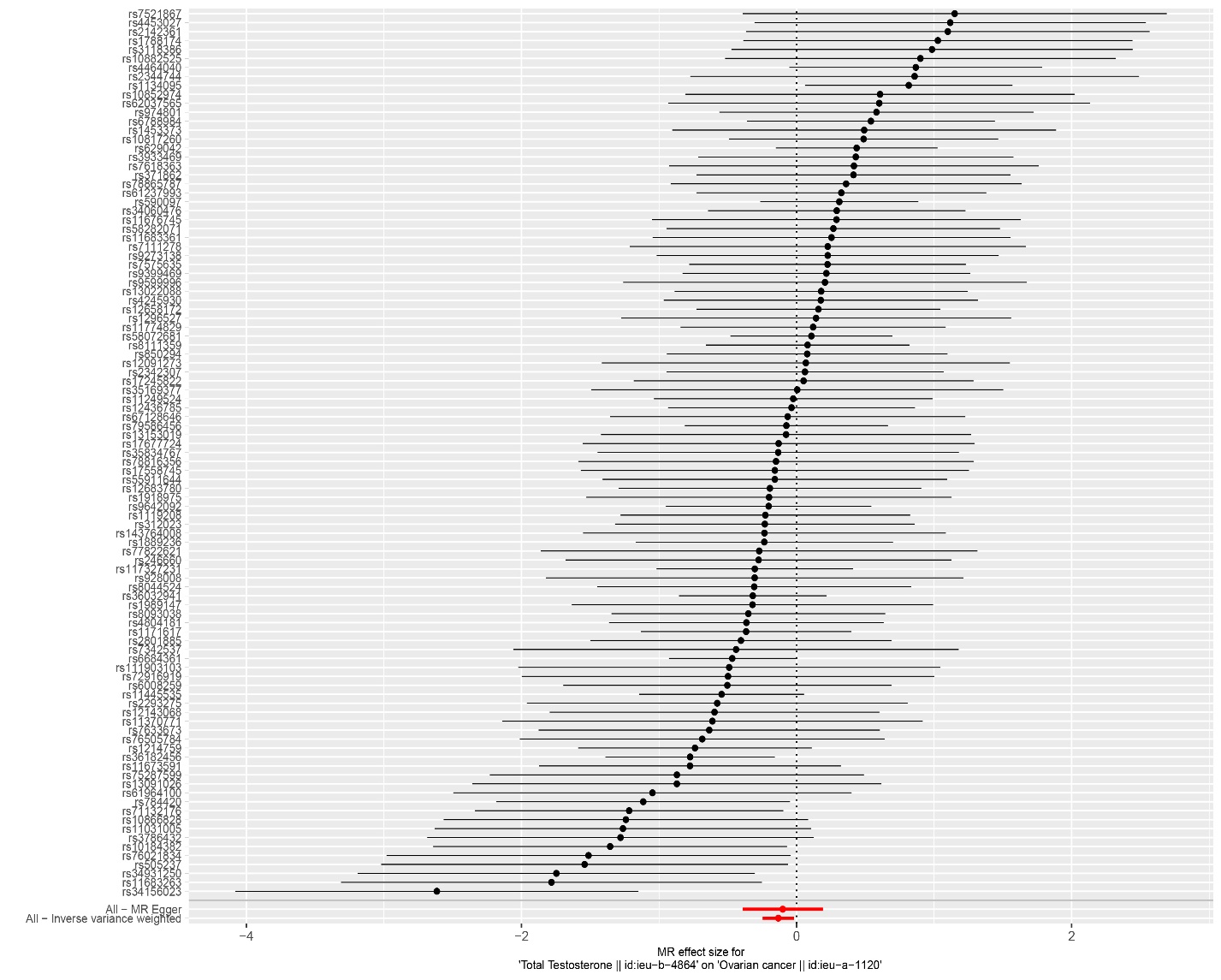


Supplementary Figure.9 Forest plot of the causal effect of total testosterone (TT) on ovarian cancer.


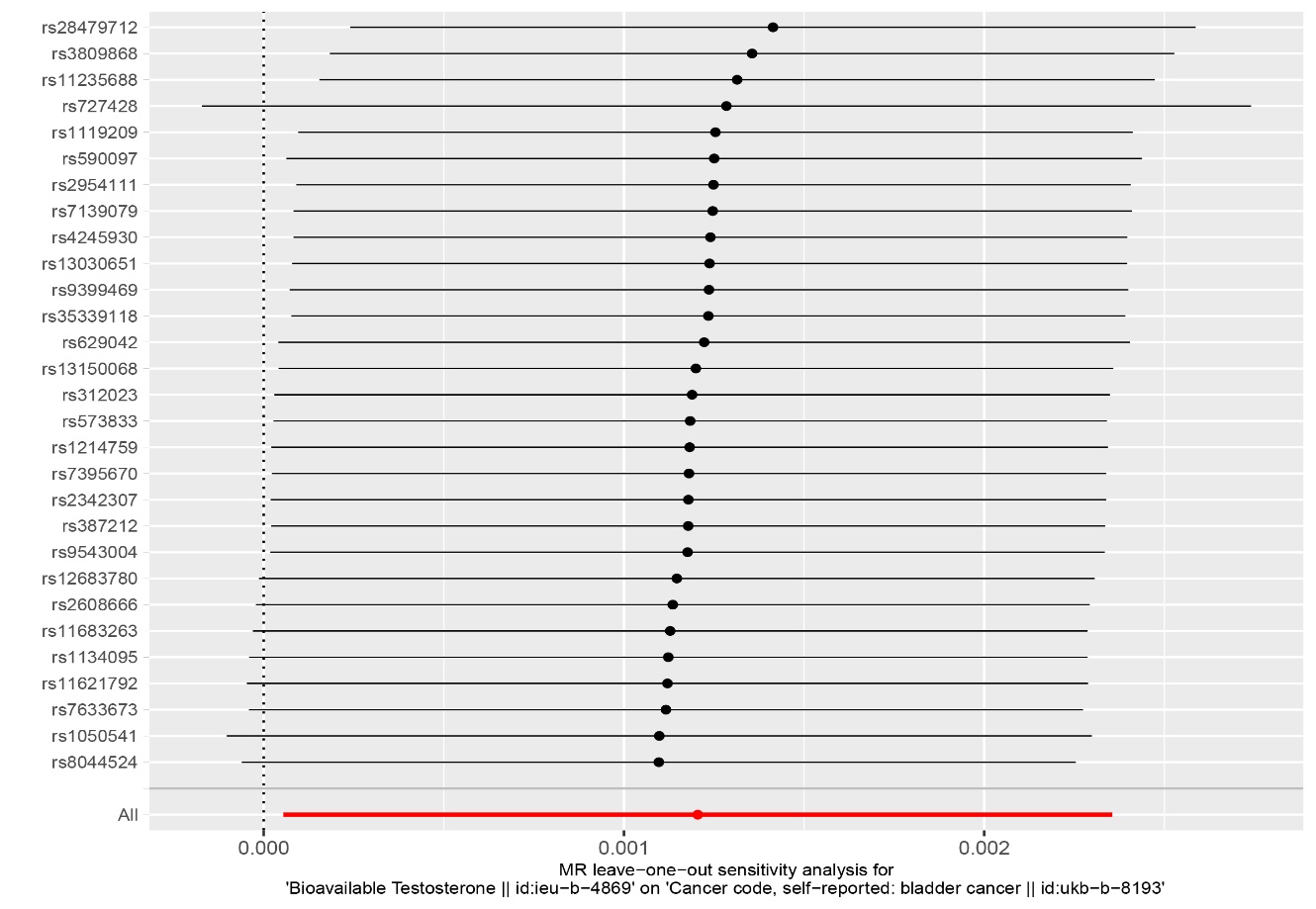


Supplementary Figure.10 Leave-one-out analysis of bioavailable testosterone (BT) on bladder cancer.


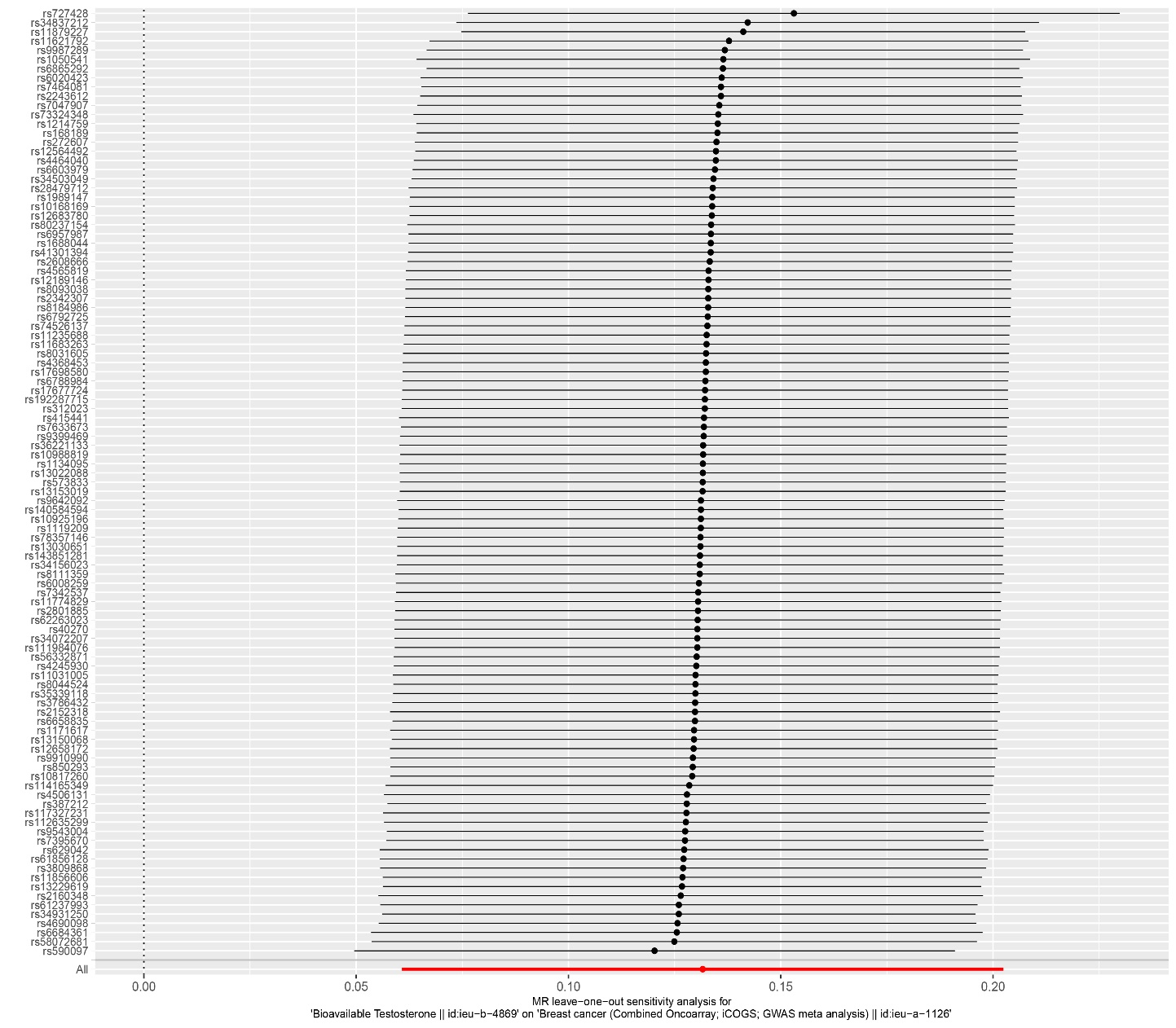


Supplementary Figure.11 Leave-one-out analysis of bioavailable testosterone (BT) on breast cancer.


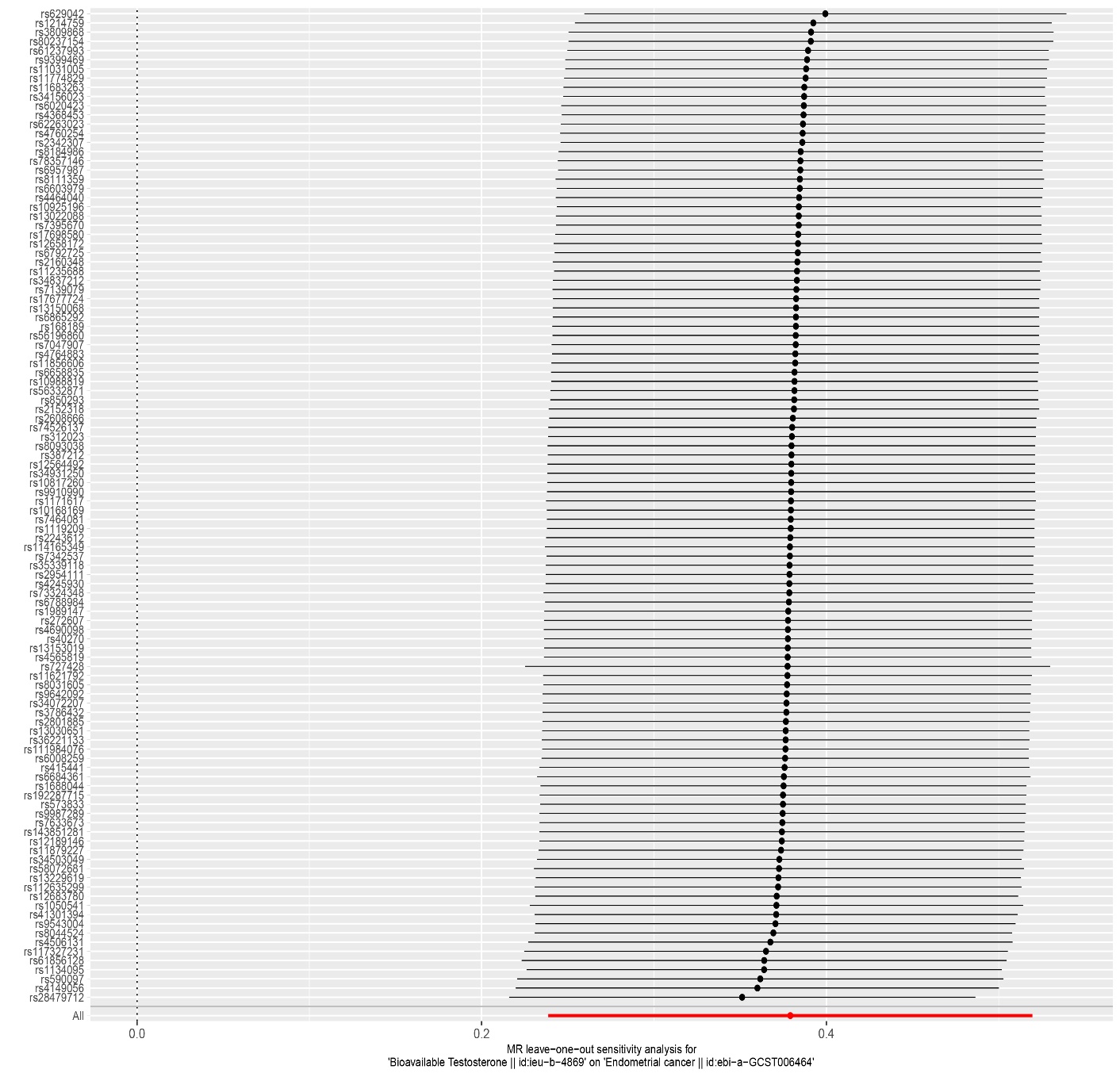


Supplementary Figure.12 Leave-one-out analysis of bioavailable testosterone (BT) on endometrial cancer.


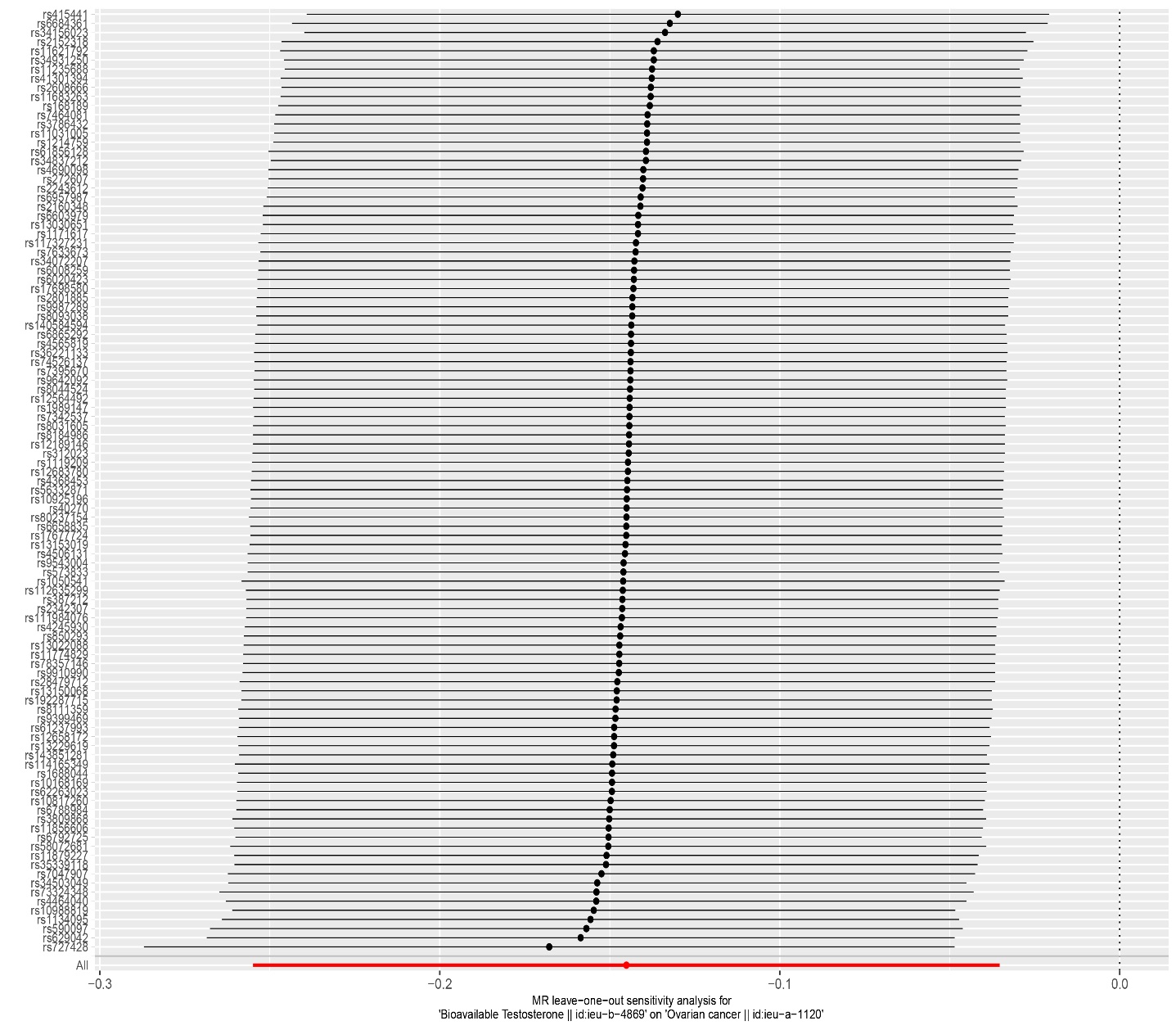


Supplementary Figure.13 Leave-one-out analysis of bioavailable testosterone (BT) on ovarian cancer.


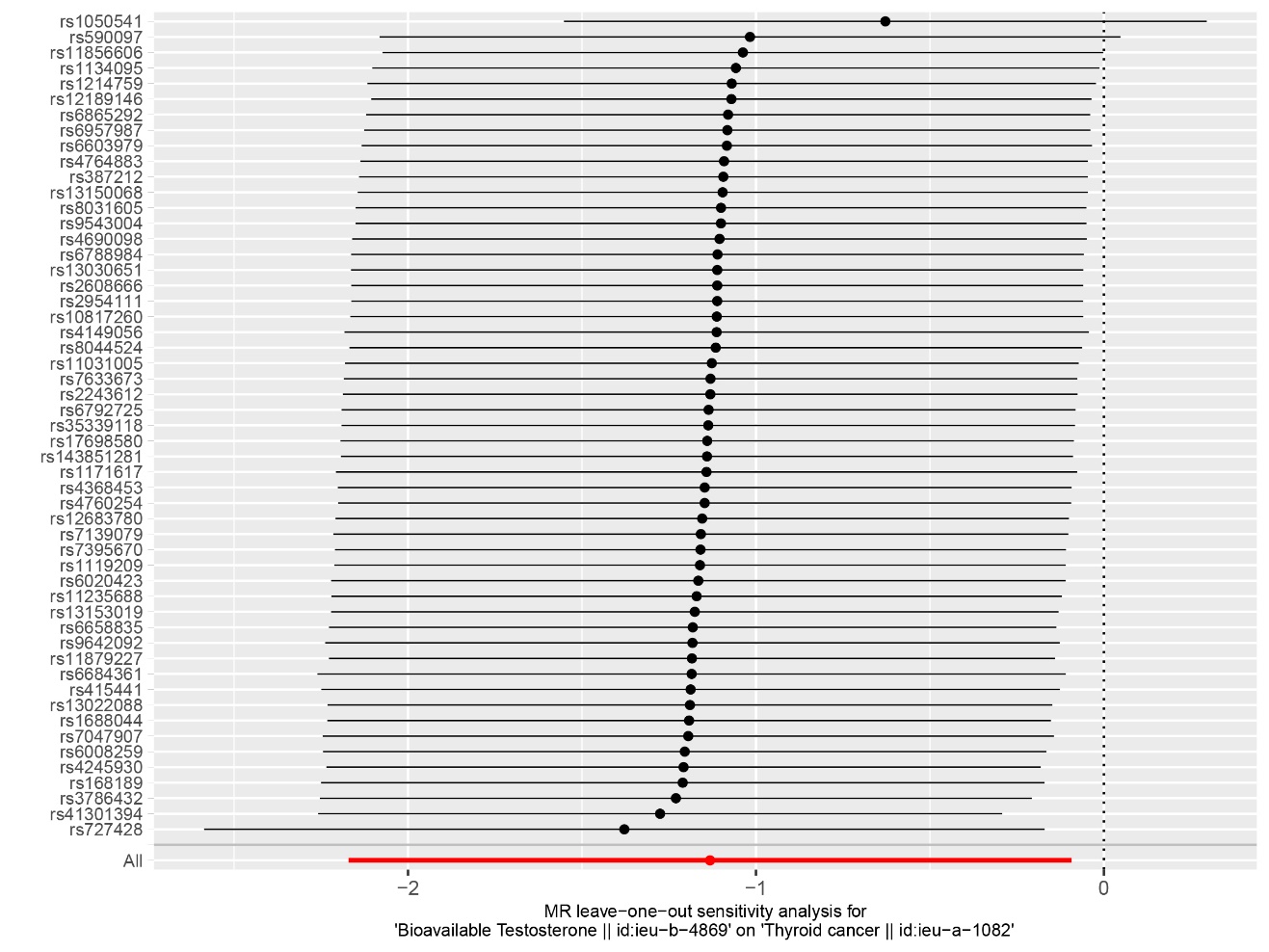


Supplementary Figure.14 Leave-one-out analysis of bioavailable testosterone (BT) on thyroid cancer.


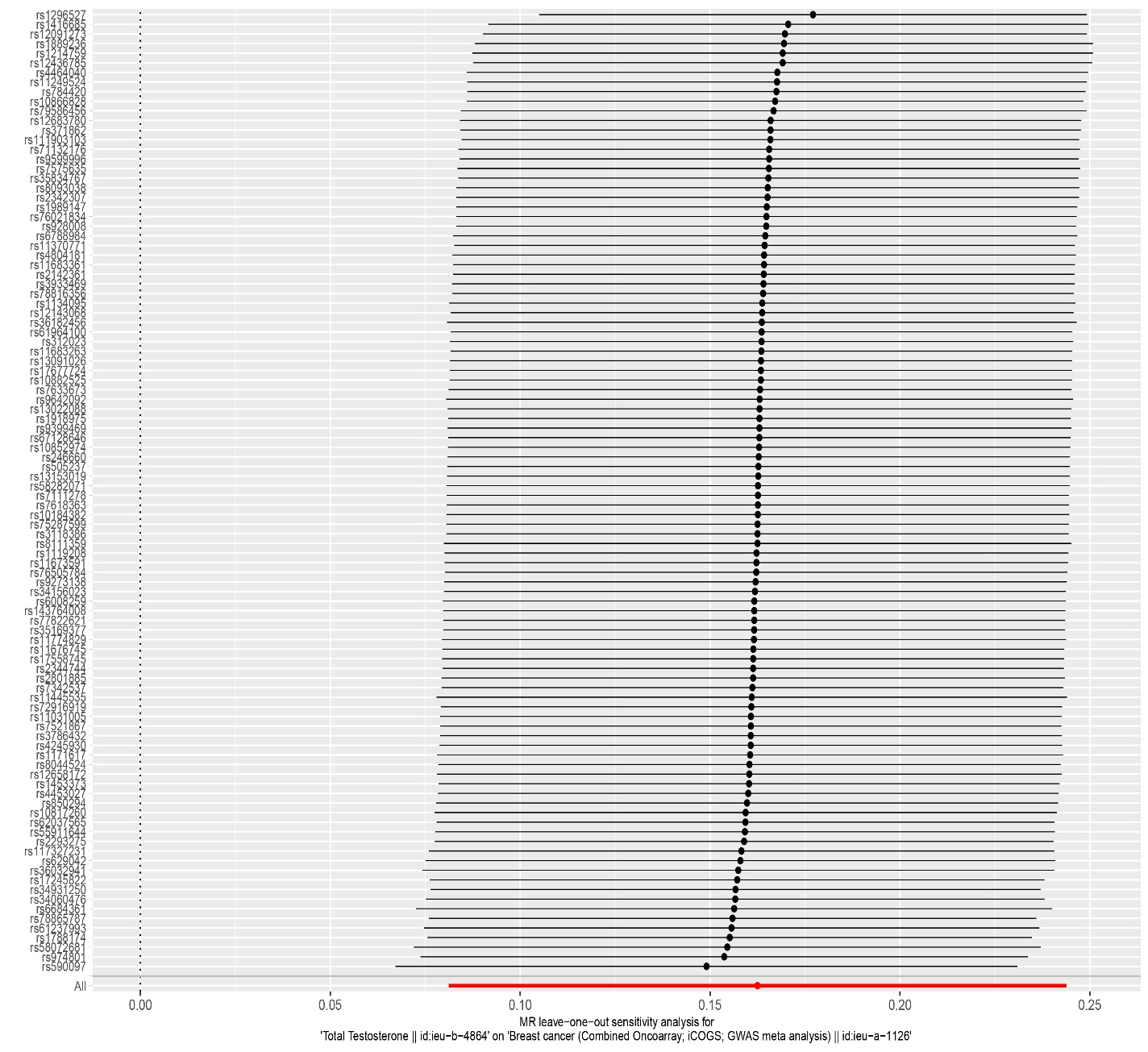


Supplementary Figure.15 Leave-one-out analysis of total testosterone (TT) on breast cancer.


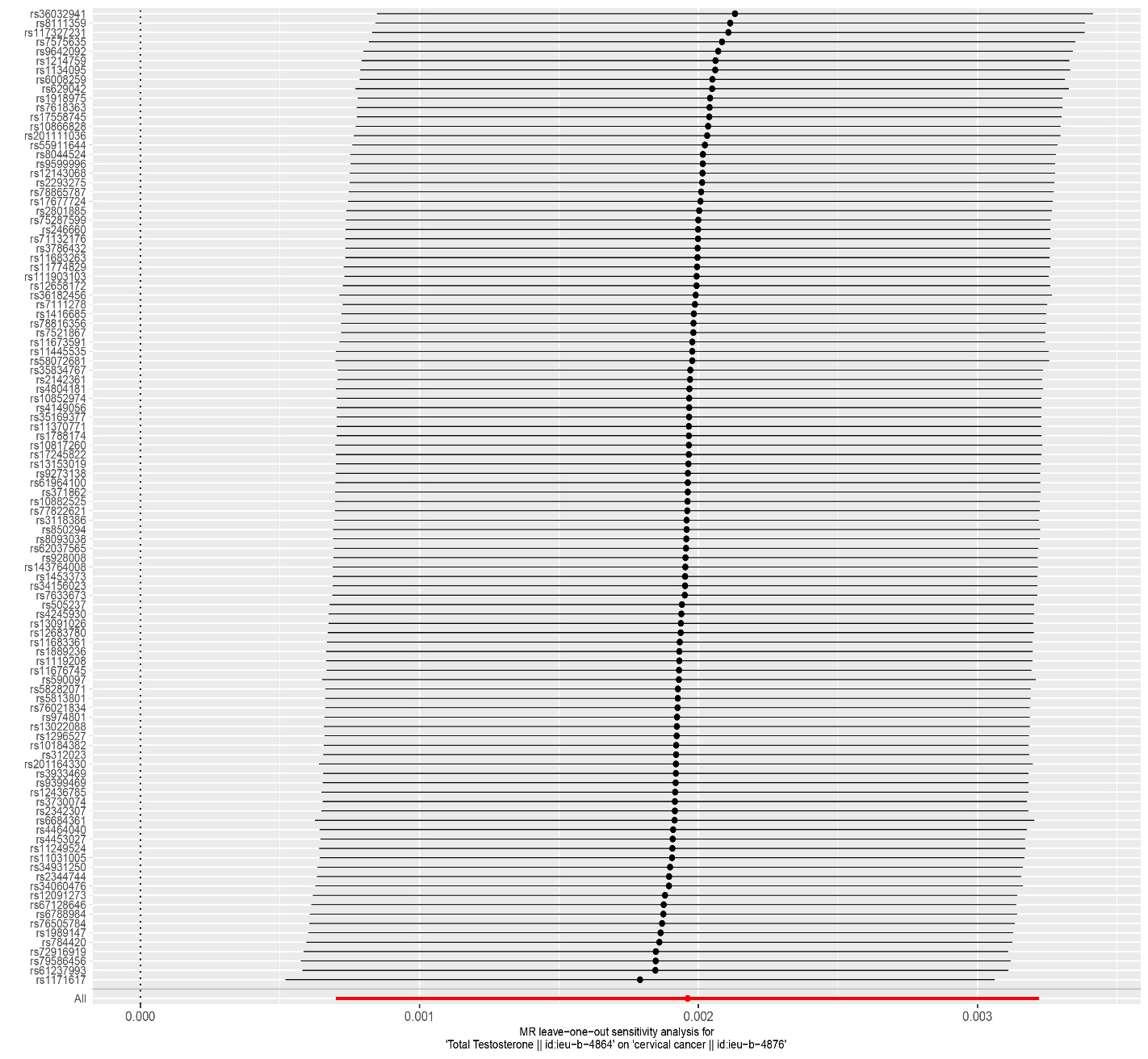


Supplementary Figure.16 Leave-one-out analysis of total testosterone (TT) on cervical cancer.


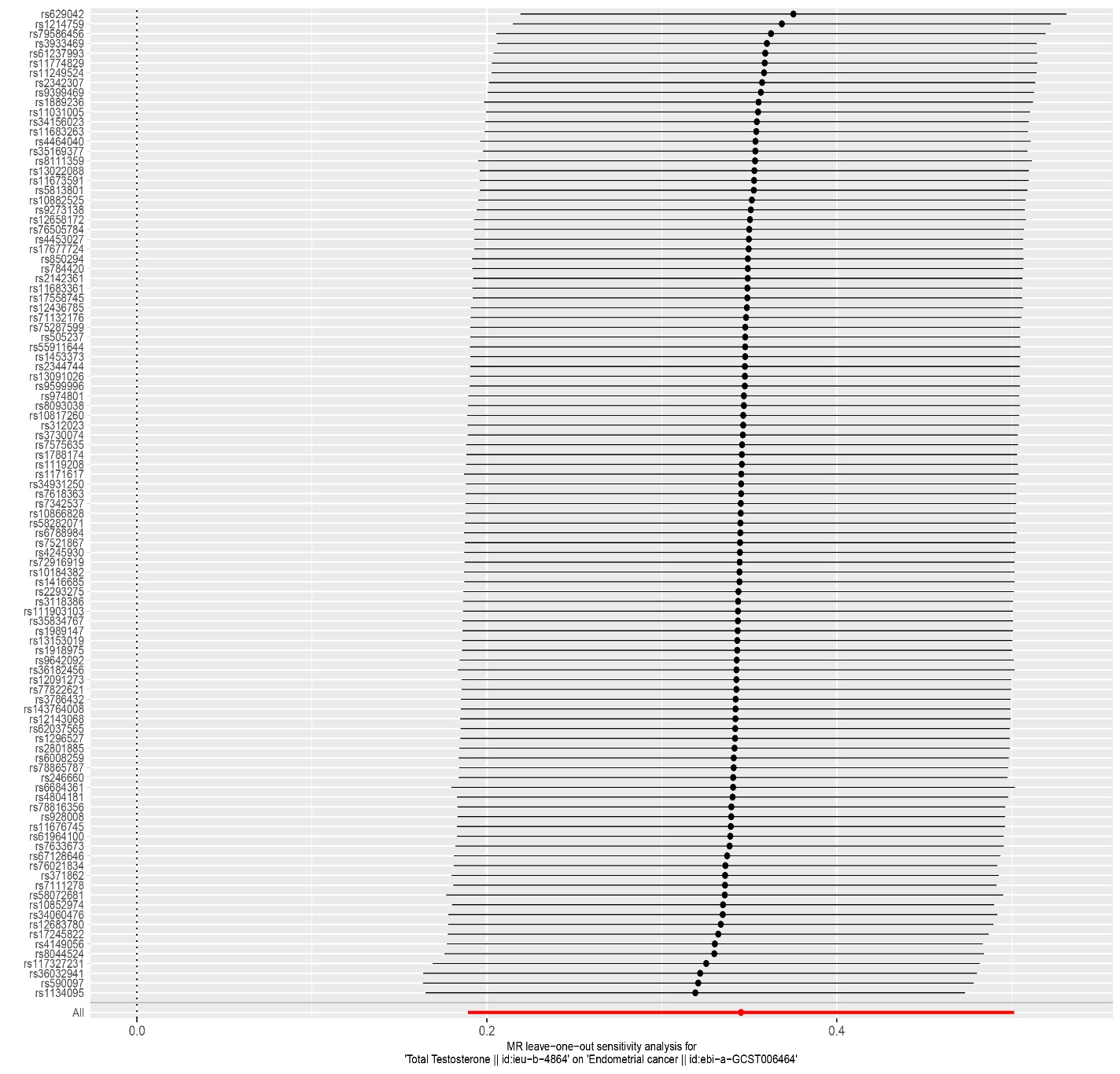


Supplementary Figure.17 Leave-one-out analysis of total testosterone (TT) on endometrial cancer.


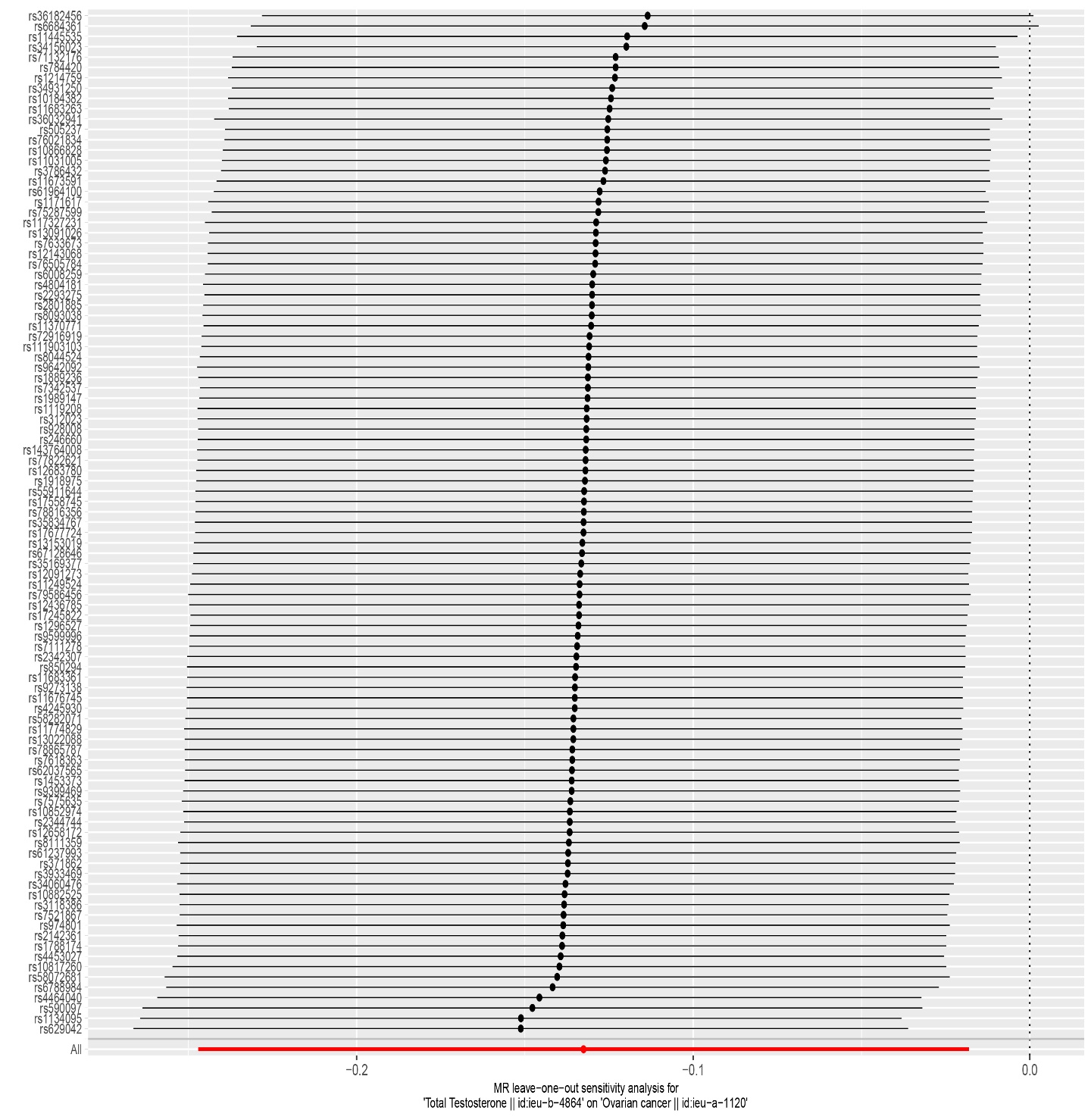


Supplementary Figure.18 Leave-one-out analysis of total testosterone (TT) on ovarian cancer.
